# A prototype integrated approach for sustainable treatment of organic dyes system using ZnO–CuO–AgO heterostructure as photocatalyst

**DOI:** 10.1038/s41598-025-29850-1

**Published:** 2025-12-11

**Authors:** Muni Raj Maurya, Sumalatha Bonthula, John-John Cabibihan, Alanood Alsafri, Omar Al Sakka Amini, Noora Noora, Kishor Kumar Sadasivuni

**Affiliations:** 1 Center for Advanced Materials, Qatar University, PO Box 2713, Doha, Qatar; 2 Department of Mechanical and Industrial Engineering, Qatar University, PO Box 2713, Doha, Qatar; 3 College of Engineering, Qatar University, PO Box 2713, Doha, Qatar

**Keywords:** Metal oxide, Heterostructure, Photodegradation, Quaternary dye system, Photocatalysis, Environmental, health and safety issues

## Abstract

Water contamination by dyes poses a significant concern for both human health and the environment. Adsorption-based, coagulation, and membrane separation methods have been investigated for the degradation or removal of dyes. However, the use of metal oxide photocatalysts has emerged as a promising approach to address this issue. The efficiency of single metal oxide is limited by high electron-hole pair recombination rates and inefficient absorption of visible light. In the present study, we investigate the ZnO–CuO–AgO photocatalyst for enhanced photodegradation of organic dyes (azo carmine (AC), cresol red (CR), indigo carmine (IC), and neutral red (NR)). ZnO–CuO–AgO nanocomposite is prepared via the hydrothermal method. The structure of the nanocomposite is analyzed by XRD and FTIR spectroscopy. The morphology of the nanocomposite is examined by TEM analysis. Dye degradation is examined using UV-visible spectroscopy, and the degradation efficiency is estimated by analyzing the absorbance curve. Photodegradation studies of AC, CR, IC, and NR dyes show optimal performance at pH 7 with a photocatalyst loading of 0.2 gL^− 1^ for 25 ppm dye solutions. The degradation percentages of AC, CR, IC, and NR are 94.7%, 80.65%, 97.15%, and 60.16%, respectively. The photodegradation of quaternary dye solution by the nanocomposite exhibited a degradation efficiency of 86.72%. In addition, turbine-based and cloth-based prototypes decorated with ZnO–CuO–AgO exhibit high degradation efficiencies of 91.41% and 88.58%, respectively.

**Supplementary Information:**

The online version contains supplementary material available at 10.1038/s41598-025-29850-1.

## Introduction

The dyeing industry, vital for textile, leather, and paper production, relies on a diverse range of synthetic and natural dyes^1^. The release of untreated or inadequately treated dye wastewater into water bodies not only affects aquatic ecosystems but also raises concerns about its impact on human health^1^. Some organic dyes exhibit persistence in the environment, resisting degradation over time. This persistence can lead to bioaccumulation in living organisms, potentially impacting the food chain and posing risks to aquatic and terrestrial ecosystems. Treatment of dye-contaminated wastewater has become a focal point in environmental engineering, necessitating the development of efficient and sustainable technologies. Traditional wastewater treatment methods, such as physical and chemical treatments, are often insufficient for complete dye removal. Consequently, advanced treatment processes, including biological treatments^2^, adsorption^3^, ion exchange removal^4^, membrane filtration^5^, catalytic reduction^6^, and photocatalytic degradation technologies^7^, have gained prominence due to their effectiveness in reducing dye concentrations and minimizing environmental impact. Biological treatment methods harness the metabolic activities of microorganisms to break down or transform dye molecules into less harmful byproducts. Adsorption technologies, utilizing materials like activated carbon, zeolites, or agricultural waste, can selectively capture dye molecules from wastewater. However, drawbacks such as low adsorption efficacy, incomplete pollutant depletion, and poor mechanical stability of adsorbents pose challenges to significant pollutant removal^3^. In recent years, photodegradation has gained significant popularity. Photodegradation offers advantages over traditional water treatment technologies. For instance, active photocatalysts can completely degrade organic pollutants within a few hours^6^. Moreover, the process avoids the formation of toxic byproducts, ensuring the complete mineralization of organic pollutants into byproducts like carbon dioxide and water.

The charge-carrier utilization affects the generation of reactive radicals and the photodegradation efficiency^8^. Studies report that the extensive electron-hole recombination rate suppresses the potential of semiconductor metal oxides as photocatalysts^6^. Diverse strategies, such as doping and heterostructure photocatalyst formation, have been applied to improve the photodegradation efficiency^6,9^. Doping with metals or other materials reduces the band gap energy of photocatalysts, enabling the material to absorb visible light more effectively, which is crucial for activating the photocatalytic process^10^. Doping can introduce new energy levels within the band structure of the photocatalyst, facilitating the separation of electron-hole pairs^11^. The incorporation of polymeric dopants, such as chitosan^12,13^ and Polyvinylpyrrolidone^14^, can provide additional functional groups that enhance the interaction with dye molecules. This increase in active sites improves the adsorption of dyes onto the catalyst surface, which is a critical step in the degradation process. Rani et al.^15^ reported samarium-grafted carbon nitride-doped bismuth oxobromide quantum dots (Sm-g-C_3_N_4_ doped-BiOBr) as an effective catalyst for the degradation of rhodamine B (RhB) in a neutral medium. The formed heterojunctions modified the electronic properties of the materials, resulting in improved light absorption across a broader spectrum. Samarium contributes to the effective separation of charge carriers (electrons and holes) generated during the photocatalytic process, achieving the dye degradation efficiency of 91.9%. Moeen et al.^16^ reported the degradation of methylene blue dye using magnesium- and chitosan-doped tin oxide quantum dots. The study revealed that doping tin oxide with magnesium and chitosan significantly enhanced its photocatalytic and sonophotocatalytic activities. The incorporation of magnesium acted as an electron acceptor, which helped separate the electron-hole pairs, thereby achieving a dye degradation efficiency of 99% in a basic medium. Numerous studies have shown that heterostructure metal oxide composites have stronger photocatalytic activity than single metal oxide nanoparticles^6^.

Heterojunctions facilitate the separation of photogenerated electron-hole pairs. The energy band alignment in a heterojunction creates a built-in electric field at the interface. This field helps to drive the electrons and holes to opposite sides, reducing the likelihood of recombination and increasing the availability of charge carriers for the degradation reaction^17^. The combination of materials with different band gaps in heterojunctions enables the overall absorption spectrum to be broadened, enabling the photocatalyst to utilize a broader range of the solar spectrum^18^. Furthermore, heterojunctions can create new active sites at the interface of the two materials^19^. These sites can enhance the adsorption of dye molecules, which is a crucial step in the photocatalytic degradation process. The increased surface area and active sites can lead to higher degradation rates. Mahdi et al.^20^ fabricated NiV₂O₆/CeO₂ nano-heterojunction using a solution combustion method. The formation of a heterojunction between NiV₂O₆ (p-type) and CeO₂ (n-type) facilitated the effective separation of photogenerated electron-hole pairs. The synthesized nanocomposites exhibited excellent photocatalytic activity, achieving a degradation rate of 93.3% for crystal violet dye under visible light irradiation within 120 min. In another study, Chen et al.^21^ prepared a LaNiO_3_/SrCeO_3_ (p-n type) heterojunction using a co-precipitation method. The study highlights that the LaNiO_3_/SrCeO_3_ heterojunction enhances the separation of charge carriers and exhibits a lower energy band gap (1.65 eV) compared to its individual components, which resulted in an improved degradation rate of 93.5% for Methylene Blue dye under visible light.

A review article^22^ discusses the photodegradation of dyes by different morphologies of the nanomaterials and their mechanism. In another study, ZnO surface was decorated with silver (Ag) and Ag-ZnO composite displayed significant improvement photodegradation of cationic and anionic dyes^23^. A study on the photocatalytic behaviour of Ag_2_SO_4_-deposited ZnO indicates its applications in wastewater treatment and the photo remediation of indigo carmine dye^24^. Thus, it is intriguing to study the ZnO–CuO–AgO heterostructure. The junction formation is expected to reduce charge carrier recombination, and photocatalysts will exhibit significantly higher photocatalytic activity.

In the current study, ZnO–CuO–AgO heterostructure is prepared by a hydrothermal process. The hydrothermal method was chosen to synthesize the ZnO–CuO–AgO photocatalyst as it enables the controlled nucleation and growth of nanostructures, resulting in significantly improved crystalline heterostructures compared to solution-based methods^25^. It is effective for synthesizing multi-metal oxide nanocomposites, such as ZnO–CuO–AgO, as it facilitates uniform doping and strong interfacial contact between different metal oxides, which are crucial for enhancing charge separation and photocatalytic efficiency^6^. Moreover, the hydrothermal process operates at relatively low temperatures and pressures, making it cost-effective and compatible with the in-situ deposition of photocatalysts on various substrates, such as 3D-printed blades and cloth fibers, which was central to our prototype development. The effect of pH and photocatalyst loading was examined for the photodegradation of dyes. The photodegradation rate kinetics and dye degradation efficiency were examined by analyzing the photocatalyst performance. The photocatalyst is employed for the photodegradation of the quaternary dye system. In addition, 3D-printed turbine-based and cloth-based prototypes decorated with ZnO–CuO–AgO nanocomposite are developed and investigated for the photodegradation of the quaternary dye solution.

The integration of a ZnO–CuO–AgO heterostructure photocatalyst into practical, scalable prototypes-specifically, 3D-printed turbine-based and cloth-based systems, highlights the efficient photodegradation of quaternary organic dye systems. Unlike conventional approaches that rely on powder-based catalysts, this study demonstrates direct deposition of the photocatalyst onto functional substrates, enabling continuous dye degradation with minimal catalyst loss and operational simplicity. The combination of multi-metal oxide synergy, visible-light responsiveness, and prototype engineering for real-world application presents a novel and sustainable approach to wastewater treatment.

## Materials and methods

### Materials

Azo carmine (AR), Cresol red (CR), Indigo carmine (IC), and Neutral red (NR) were purchased from Merck. Zinc acetate dihydrate (Zn(CH_3_COO)_2_·2H_2_O, ≥ 98%), Sodium hydroxide pellets (NaOH, > 97%), Copper (II) acetate monohydrate (Cu(CH_3_COO)_2_·H_2_O, ≥ 98% ), Hydrochloric acid (HCl, 37%), Silver nitrate (AgNO_3_, > 99%) were obtained from Merck. The chemicals used are all reagent grade and weren’t further purified before utilization. The experiments were conducted utilizing Millipore Milli-Q double-distilled water.

### Synthesis of ZnO–CuO–AgO

Hydrothermal method was used to synthesize the ZnO–CuO–AgO composite. 5.504 g of anhydrous zinc acetate was dissolved in 100 mL of deionized (DI) water and stirred for 20 min. Separately, 3.75 g of NaOH were dissolved in 100 mL of double-distilled water and stirred for 15 min. The NaOH solution was added dropwise into the zinc acetate solution and stirred for 30 min at 60 °C, forming a white, gelatinous precipitate. Following this, a solution containing 5.448 g of anhydrous copper acetate and 0.509 g of silver nitrate, dissolved in 100 mL of DI water, was slowly added over 10 min to the reaction mixture while maintaining a temperature of 80 °C for one hour with constant stirring. The obtained total volume of the solution was 300 ml with anhydrous zinc acetate (0.1 mol L^−1^), anhydrous copper acetate (0.1mol L^-1^) and silver nitrate (0.01 mol L^−1^) mixed in the molar ratio of 10:10:1^26,27^. The obtained mixture was subsequently sealed in an autoclave, and hydrothermal synthesis was carried out at 180 °C for 6 h. The resulting nanocomposite was centrifuged at 4000 rpm for 30 min and washed thoroughly with distilled water and ethanol five times to remove any residue. Finally, the product was dried at 80 °C for 1 h, followed by annealing at 400 °C for 4 h. The annealing temperature of 400 °C promotes strong interfacial bonding between different metal oxides in heterostructures, enhancing charge separation efficiency and thus improving photocatalytic performance^28^. Temperatures significantly below 400 °C may result in incomplete crystallization, while higher temperatures risk agglomeration and loss of surface area, which are detrimental to photocatalysis.

### 3D-printed turbine decorated with ZnO–CuO–AgO

The turbine blades and the rotating body were fabricated separately using a DLP 3D printer. Following the preparation of the ZnO–CuO–AgO composite mixture, the 3D-printed turbine blades were immersed in the solution and enclosed within an autoclave. Hydrothermal synthesis was then conducted at 180 °C for 6 h. Subsequently, the ZnO–CuO–AgO decorated blades were rinsed five times with DI water and were left to dry at 80 °C overnight. Finally, the nanocomposite-decorated blades were affixed into the slots of the rotating body, completing the assembly of the turbine configuration.

### Cotton fiber cloth decorated with ZnO–CuO–AgO

The cotton fiber cloth was cleaned using ethanol to remove organic contaminants, surface oils, and particulate matter that may be present on the fabric surface. This pre-treatment step will ensure better adhesion and uniform deposition of the ZnO–CuO–AgO nanocomposite during the hydrothermal synthesis. The cleaned cloth was dried at 60 °C for 6 h to remove the ethanol. Subsequently, the ZnO–CuO–AgO composite-forming mixture was prepared according to the method outlined in “Synthesis of ZnO–CuO–AgO” section. The cleaned cotton cloth was then immersed in the solution and enclosed in an autoclave. Hydrothermal synthesis was conducted at 180 °C for 6 h. After synthesis, the ZnO–CuO–AgO decorated cloth underwent multiple rinses with DI water and ethanol, then drying at 80 °C overnight.

### Photodegradation of dyes

The photocatalytic performance of ZnO–CuO–AgO was studied in aqueous AC, CR, IC, NR, and quaternary dye (AC, CR, IC, NR) solutions under sunlight. Experiments were performed under consistent settings on a sunny day between 11:00 and 14:00, with an exposure duration of 100 min. For the photodegradation process, 1 mg of ZnO–CuO–AgO composite was introduced into 5 mL of 25 ppm dye solutions with pH values of 3, 7, and 9. The 25 ppm concentration of dye was selected, as it provided a balanced concentration that was high enough to allow for accurate spectrophotometric detection of degradation over time, while avoiding saturation effects or optical interference during photocatalytic testing. This concentration also ensured consistency across comparative studies involving different dyes and catalyst prototypes within our investigation^29^. The photocatalyst loading effect on the dye degradation was studied by varying the catalyst loading from 1 to 7 mg in 25 ppm of 5 mL dye solution. The photodegradation of the quaternary dye system consists of 25 ppm of each dye concentration in 5 mL of aqueous dye solution. The nanocomposite decorated turbine and cloth studies were carried out in a 200 mL aqueous quaternary dye system with each dye concentration of 25 ppm.

### Characterization

The structure and morphology of the ZnO, ZnO-CuO, and ZnO–CuO–AgO composites were examined with an X-Ray diffractometer (X’PERT-Pro MPD, PANalytical Co., Almelo, Netherlands) and TEM (FEITECNAI G2 TEM, TF20), respectively. The composite formation was confirmed with a Thermo Nicolet Nexus 670 FTIR spectrometer (KBr pellets) and an X-Ray Photoelectron Spectrometer (XPS), AXIS Ultra DLD. The Brunauer-Emmett-Teller (BET) (NOVA 2200e) specific surface area was determined by nitrogen adsorption-desorption isotherm. The band gap of the composite and the effect of dye concentration on photodegradation were characterized using a UV-vis spectrophotometer (Biochrom Ltd, Cambridge, United Kingdom). The morphology of the ZnO–CuO–AgO composite deposited on the turbine and cotton fiber cloth was studied by scanning electron microscopy (SEM, Hitachi S-4800, Hitachi, Tokyo, Japan). The photoluminescence (PL) spectrum of the nanocomposite was obtained through a fluorescence spectrophotometer (Perkin Elmer LS-55).

## Results and discussion

### Structure of the nanocomposite

**Fig. 1:**
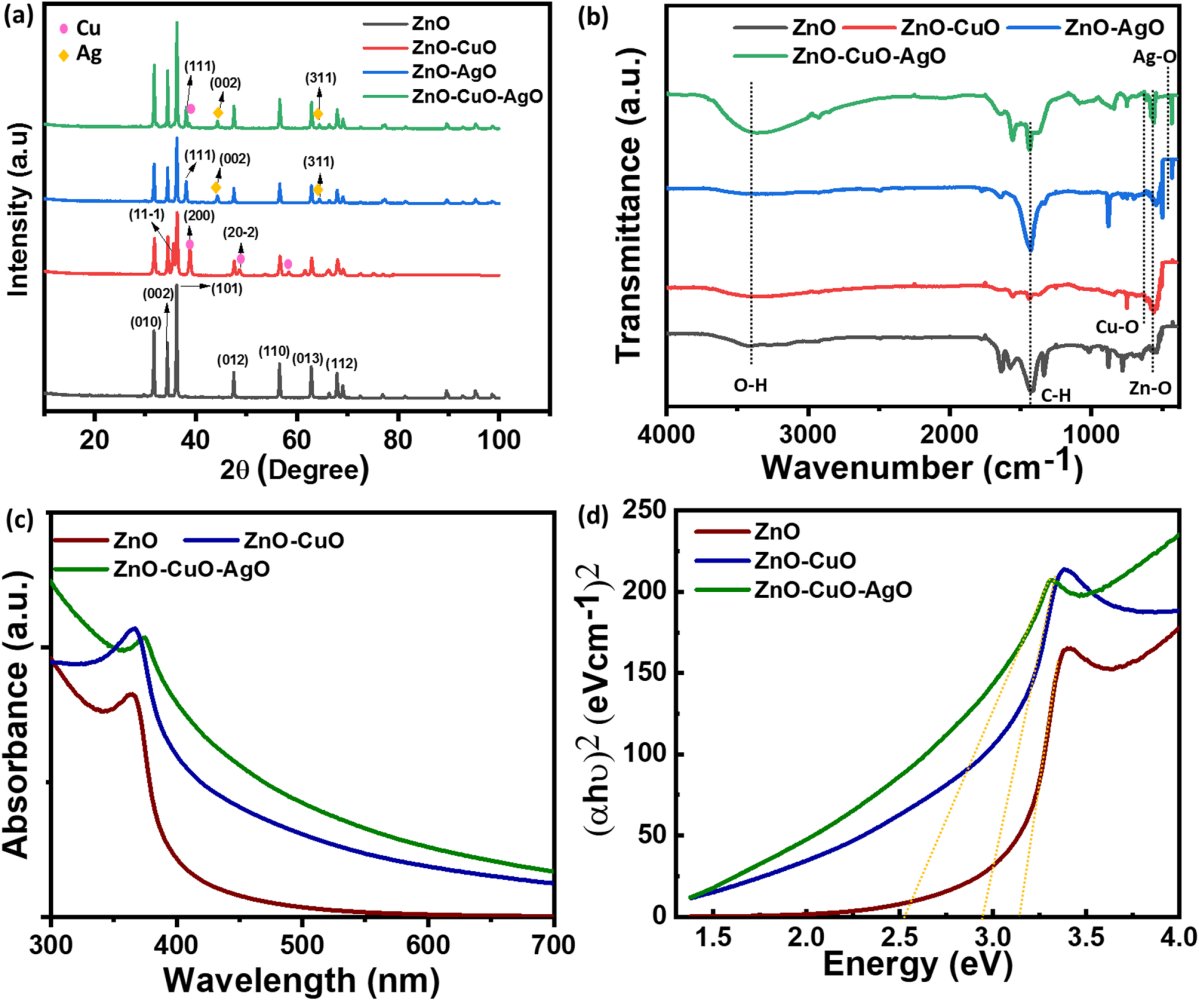
Structural analysis of the synthesized nanomaterial. (**a**) XRD plot of the nanocomposite. (**b**) FTIR spectra of the nanocomposite. (**c**) UV-visible absorbance curve of the nanocomposite. (**d**) Tauc plot, presenting the band gap of the nanocomposite.

The structure of the synthesized nanomaterials was analyzed using X-ray diffraction (XRD). Figure 1a illustrates the XRD spectra for pure ZnO, ZnO-CuO, ZnO-AgO, and ZnO–CuO–AgO nanocomposites synthesized via the hydrothermal method. The observed diffraction peaks at 2θ values of 31.71°, 36.21°, 56.55°, and 67.90° corresponded to the (010), (101), (110), and (112) planes, respectively, consistent with the hexagonal wurtzite structure of ZnO, in agreement with standard data (JCPDS-89-0510). Notably, all samples displayed a high orientation along the (101) reflection plane. In the XRD plot of ZnO-CuO, the appearance of new peaks at 2θ values of 38.72° and 58.37° confirmed the presence of copper and indicated the formation of a heterostructure. Similarly, additional diffraction peaks observed in the ZnO-AgO XRD spectra at 2θ values of 44.26° and 64.40° could be attributed to the (002) and (311) planes of silver. The presence of copper and silver peaks at 2θ values of 38.72°, 44.29°, and 64.44°, respectively, further confirmed the formation of the ZnO–CuO–AgO composite heterostructure, as depicted in Fig. 1a^30,31^. The crystallinity of the ZnO–CuO–AgO composite was estimated by Eq. (1)^32^.

1 $${\text{Crystallinity}}\, (\%) = 100 \times\:\:\frac{{A}_{c}}{{A}_{t}}$$

where $\:{A}_{c}$ is an area under the crystalline peaks and $\rm{Grain (crystallite) size (G.S.)} =0.9 \lambda / \beta \rm{cos} \theta$ is the total area. The crystallinity of the ZnO–CuO–AgO composite was calculated as 89.05%.

The grain size of ZnO–CuO–AgO nanocomposite was calculated by using the Scherrer relation given in Eq. (2)^12^.

where G.S is the grain size of the ZnO–CuO–AgO composite, $\:\beta\:$ is the full-width at half-maximum and $\:\lambda\:$ is wavelength in nm. The average grain size of the ZnO–CuO–AgO composite was calculated as 34.5 nm.

The specific surface area distribution of ZnO–CuO–AgO was estimated using BET, and the adsorption-desorption isotherm graph is shown in the supplementary information Fig. S1a. The nanocomposite exhibits a Type IV BET isotherm with H3-type hysteresis, which is characteristic of mesoporous materials formed by agglomeration of nanoparticles. The specific surface area of ZnO–CuO–AgO was 24.92 m^2^g^− 1^. The pore size of ZnO–CuO–AgO was approximately 2.8 nm, as shown in the supplementary information Fig. S1b. The high surface will result in the availability of more active sites, which could enhance the photocatalytic activity of nanocomposite.

The vibration behavior of the synthesized nanomaterials was analyzed by FTIR spectroscopy. Typical infrared spectra of ZnO, ZnO–CuO, ZnO–AgO, and ZnO–CuO–AgO are presented in Fig. 1b. For all samples, the broad bands near 3000–3800 cm^− 1^ with a peak centred at nearly 3450 cm^− 1^ were attributed to the O-H group stretching on the surface of ZnO, ZnO–CuO, ZnO–AgO, and ZnO–CuO–AgO catalyst. The intensity and number of peaks observed in ZnO were diminished in ZnO-CuO, ZnO-AgO, and ZnO–CuO–AgO catalysts, indicating the formation of new bonds in the composite materials. Peaks between 400 cm^− 1^ to 550 cm^− 1^ were assigned to the meta-oxygen stretching mode. In the FTIR spectra of ZnO–CuO–AgO, peaks at 546 cm^− 1^, 623 cm^− 1^, and 432 cm^− 1^were observed, corresponding to Zn-O, CuO, and AgO stretching, respectively, further indicating the formation of a heterostructure^30,33^. Figure 1c displays the UV-visible absorbance spectra of ZnO, ZnO-CuO, and ZnO–CuO–AgO composites. Notably, a red shift in the peak was observed with the formation of the heterostructure in ZnO-CuO and ZnO–CuO–AgO, compared to the ZnO absorbance curve. The band gap of the nanocomposites was determined using the Tauc plot, as depicted in Fig. 1d^34^. ZnO, ZnO–CuO, and ZnO–CuO–AgO exhibited band gaps of 3.2 eV, 2.9 eV, and 2.57 eV, respectively. A decrease in the band gap towards the visible region was noted with the heterostructure formation. The reduction in the band gap after incorporating CuO and AgO into ZnO results from heterojunction formation, orbital hybridization, and interfacial charge transfer among the metal oxides. ZnO, with a direct band gap of ~ 3.2 eV, primarily absorbs UV light. Coupling with CuO (Eg ≈ 1.55 eV)^35^ and AgO (Eg ≈ 1.6 eV)^36^ forms p–n heterojunctions that introduce intermediate energy levels between the valence band (VB) and conduction band (CB), narrowing the effective band gap to ~ 2.57 eV^9^.

**Fig. 2:**
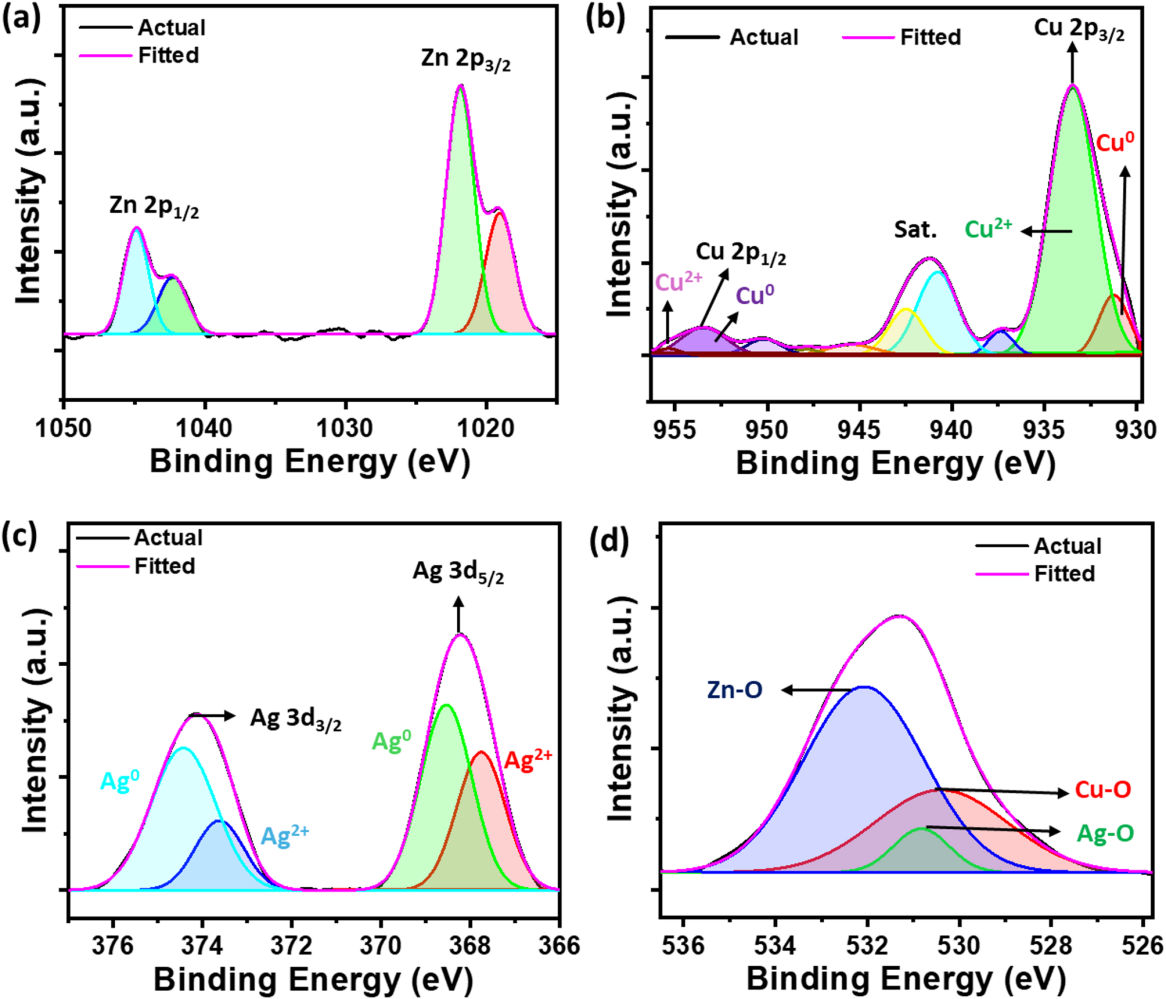
High-resolution XPS spectra of (**a**) Zn 2P. (**b**) Cu 2p. (**c**) Ag 3d. (**d**) O 1s.

Figure 2 presents the XPS analysis of the ZnO–CuO–AgO. The ZnO XPS spectrum revealed peaks at 1021.8 eV and 1044.8 eV, indicating Zn 2P_3/2_ and Zn 2P_1/2_, respectively, as shown in Fig. 2a^27,37^. In the XPS spectrum of CuO, the peak at 933.47 eV and 953.47 eV with a separation energy of 20 eV corresponds to Cu 2p_3/2_ and Cu 2p_1/2_, characteristic of Cu^2+^ ions (see Fig. 2b)^38^. The satellite peaks at 940.79 eV and 942.48 eV originate from the Cu–O bonds and confirm the existence of CuO in the composite^39^. The high-resolution XPS spectrum of Ag 3d is presented in Fig. 2c. The Ag 3d doublet showed an asymmetrically shaped peak with a binding energy of 368.2 eV (Ag 3d_5/2_) and 374.2 eV (Ag 3d_3/2_), confirming the presence of AgO and Ag^40^. The deconvolution of the spectra showed a peak at 368.53 eV and 374.42 eV, corresponding to metallic Ag^40^. The deconvoluted peak with lower binding energy at 367.78 eV and 373.62 eV indicates the presence of an Ag^2+^ state^40^. The XPS spectrum of O 1s (see Fig. 2d) consists of three peaks at 530.4 eV, 530.83 eV and 532.07 eV. The peak at 530.4 eV originates from Cu–O bonds^38^, the peak centred at 530.83 eV can be assigned to Ag–O bonds^40^, and the peaks at 532.07 eV indicate the formation of Zn-O bonds^27^. The wide XPS spectrum of ZnO–CuO–AgO is presented in the supplementary information Fig. S2.

### Morphology of the nanocomposite

**Fig. 3:**
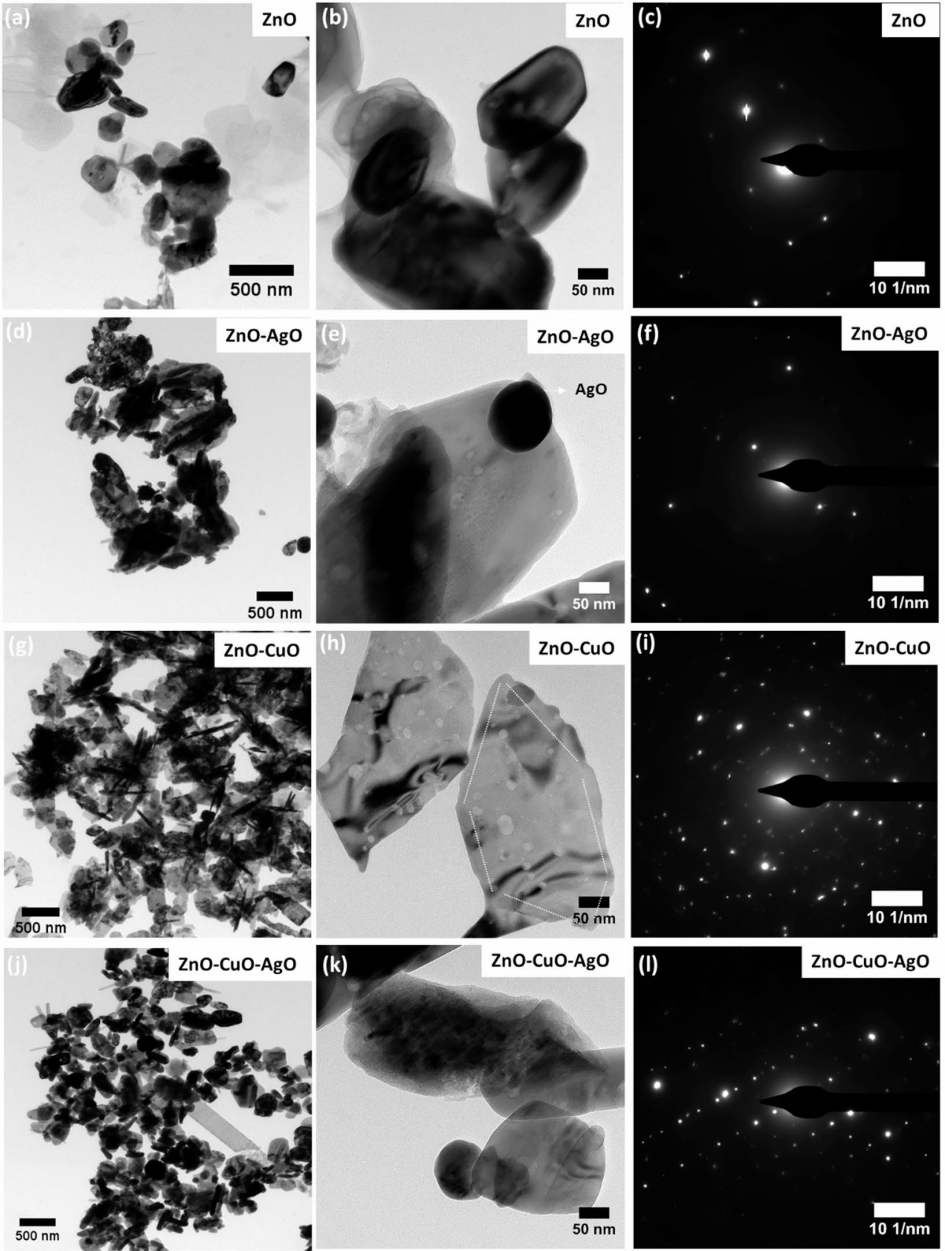
TEM image and SAED pattern of the synthesized nanocomposite. (**a**–**c**) ZnO. (**d**–**f**) ZnO–AgO. (**g**–**i**) ZnO–CuO. (**j**–**l**) ZnO–CuO–AgO.

In Fig. 3a, the ZnO sample shows disc-shaped nanoparticles. The magnified TEM image in Fig. 3b shows the smooth ZnO surface. The corresponding SAED pattern in Fig. 3c exhibits bright diffraction spots, confirming the formation of crystalline hexagonal wurtzite ZnO. The ZnO-AgO nanocomposite exhibits slightly aggregated lamellar and elongated particles, as shown in Fig. 3d. The magnified TEM image in Fig. 3e shows the growth of spherical AgO nanoparticles on the ZnO surface. The SAED pattern in Fig. 3f still shows distinct diffraction dots, indicating the crystalline nature of ZnO-AgO. The TEM image of ZnO-CuO composite is shown in Fig. 3g. The magnified image in Fig. 3h exhibits a heterogeneous structure with hexagonal and approximate cubic lattice combinations, which indicates the fabrication of ZnO-CuO nanocomposite with hexagonal and cubic crystal lattices, respectively. The corresponding SAED pattern in Fig. 3i shows bright and sharp diffraction dots, representing the good crystalline nature of ZnO-CuO. Figure 3j shows the TEM image of ZnO–CuO–AgO nanocomposite. The zoomed-in image in Fig. 3k shows the nanocomposite with a hexagonal and cubic lattice combination, representing ZnO-CuO. The growth of AgO nanoparticles on the ZnO-CuO structure is also evident from Fig. 3k, supporting the preparation of ZnO–CuO–AgO heterostructure. Figure 3l demonstrates continuous, sharp, and bright diffraction dots, confirming the crystalline nature of ZnO–CuO–AgO nanocomposite.

### Photodegradation of organic dye

#### pH effect

**Fig. 4:**
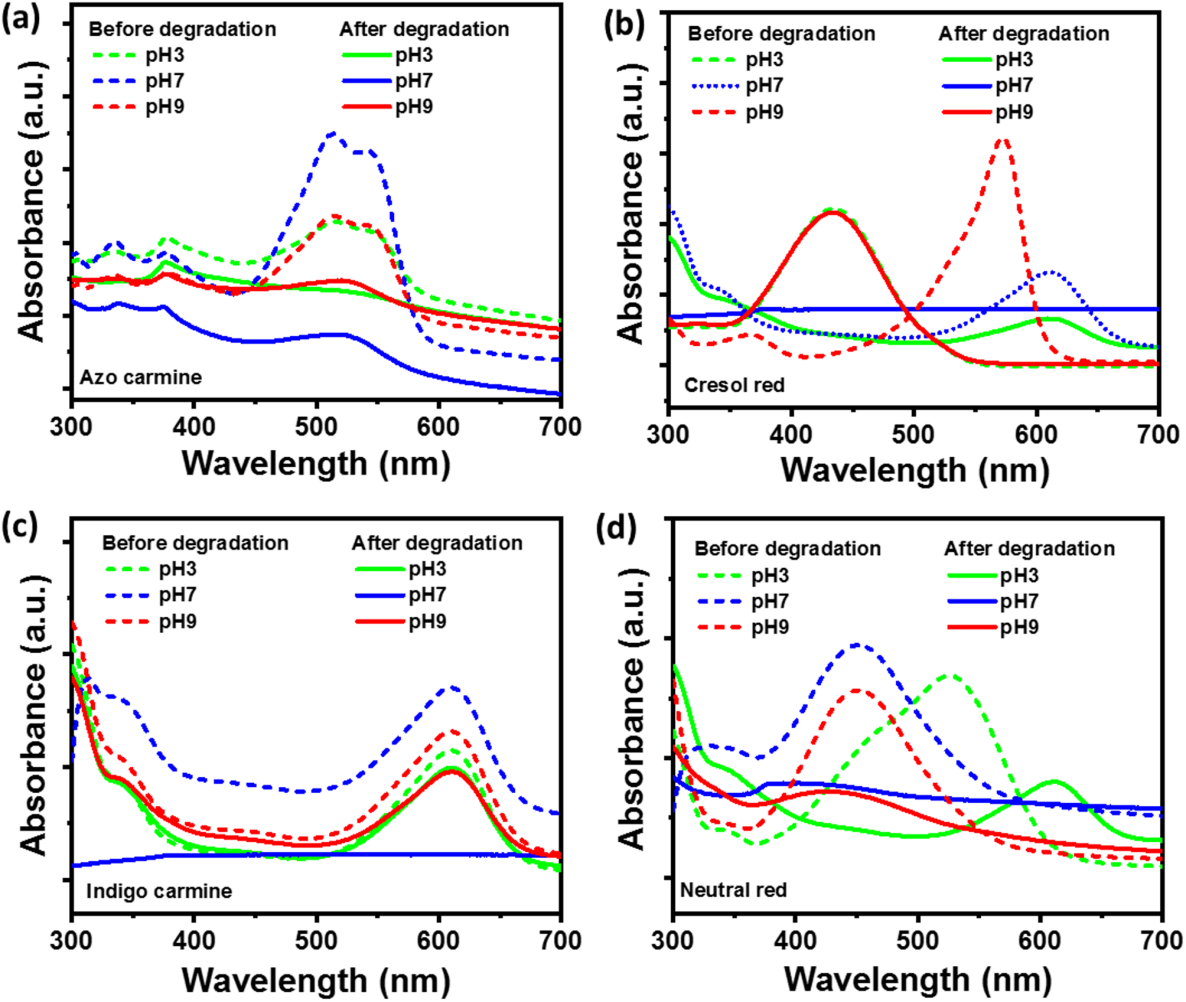
UV-Visible absorbance spectra before and after photodegradation of photocatalyst loaded dyes solution at different pH. (**a**) Azo carmine. (**b**) Cresol red. (**c**) Indigo carmine. (**d**) Neutral red.

The photocatalytic degradation performance of AC, CR, IC, and NR dyes using synthesized ZnO–CuO–AgO photocatalysts was assessed in aqueous solutions at pH 3, pH 7, and pH 9. Each dye was maintained at a concentration of 25 ppm in 5 mL of aqueous solution at different pH levels, with 1 mg of ZnO–CuO–AgO added to each dye solution. The reaction setup was then exposed to direct sunlight for 100 min. Figure 4 illustrates the analysis of dye degradation using UV-visible absorbance spectra. Notably, significant quenching in the absorbance spectra of AC dye was observed in the pH 7 solution, indicating efficient dye photodegradation after 100 min, as depicted in Fig. 4a. Similarly, pH 7 aqueous solutions of CR, IC, and NR dyes exhibited enhanced photodegradation, as shown in Fig. 4b and c, and Fig. 4d, respectively. For CR in pH 9, after photodegradation, a significant blue shift in the absorbance peak is observed, as shown in Fig. 4b. In a basic medium (pH 9), CR exists mainly in its deprotonated anionic form, displaying a strong absorption peak at 572 nm due to its conjugated π→π* system. Photocatalytic degradation under visible light results in oxidative cleavage of aromatic rings, reduction in conjugation length, and formation of transient intermediate species. As the photodegradation CR proceeds, the UV-Visible band progressively shifts toward shorter wavelengths, indicating a blue shift associated with chromophore breakdown^41,42^. After complete degradation, visible absorption nearly disappears, also indicated by the decolorization of CR. However, in basic medium, excess ·OH radicals act as radical scavengers and reduce the availability of reactive species for dye n oxidation^12^. As a result, CR dye doesn’t degrade fully in basic medium, due to which a peak at ~ 435 nm is observed after 100 min of photodegradation.

Further, the variation in dye degradation efficiency observed in Fig. 4 can be attributed to the influence of pH on (i) the surface charge of the ZnO–CuO–AgO photocatalyst, (ii) the ionization state of dye molecules, and (iii) the generation of reactive oxidative species during the photodegradation process. At neutral pH, optimal degradation was observed for all dyes. This is likely due to favorable electrostatic interactions between the photocatalyst and the dye molecules, as the surface of the ZnO–CuO–AgO nanocomposite tends to be near its point of zero charge, facilitating better adsorption of both cationic and anionic dyes. Additionally, the generation of hydroxyl (·OH) and superoxide (·O₂⁻) radicals, which are the primary reactive species in photocatalysis, is more efficient under neutral conditions, enhancing degradation rates^43^. At acidic pH, excess H⁺ ions can compete with dye molecules for adsorption sites and suppress the formation of ·OH radicals, reducing degradation efficiency^43^. Conversely, at a basic pH, although more hydroxyl ions are present, which could, in principle, form more ·OH radicals, these ions may also act as radical scavengers and reduce the availability of reactive species for dye oxidation^12^. Thus, the superior photodegradation performance observed at neutral pH results from the synergistic effects of enhanced dye adsorption and optimal radical generation.

####  Photocatalyst loading effect

**Fig. 5:**
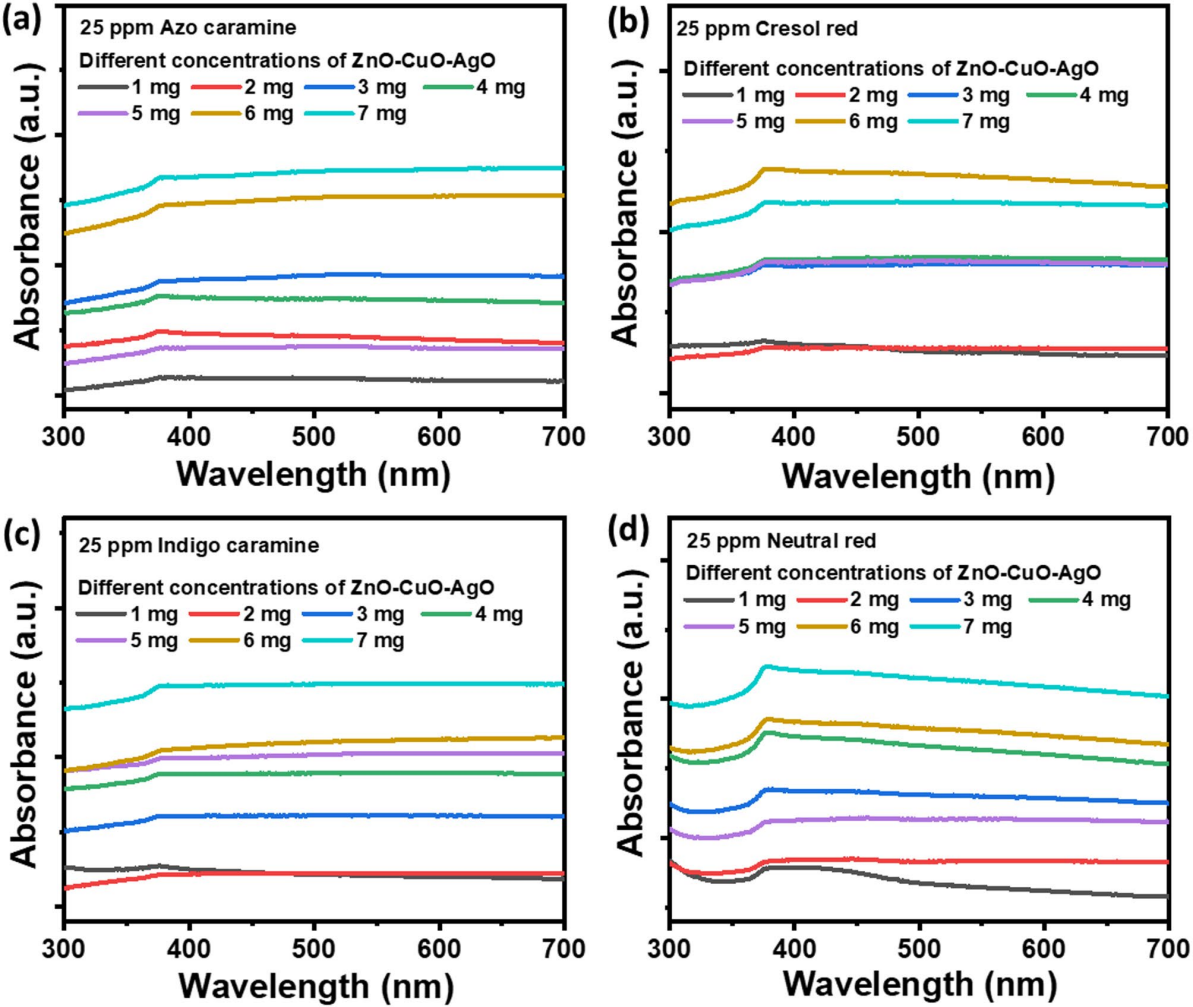
UV-visible absorbance spectra for analysing the photocatalysis loading effect of dye degradation. (**a**) Azo carmine (**b**) Cresol red. (**c**) Indigo carmine. (**d**) Neutral red.

The investigation evaluates the impact of photocatalyst loading on dye photodegradation under sunlight exposure. Solutions containing 25 ppm of AC, CR, IC, and NR dyes in 5 mL aqueous solutions at pH 7 were loaded with varying amounts (1–7 mg) of ZnO–CuO–AgO photocatalyst. Subsequently, the reaction assemblies were exposed to sunlight, and UV-visible absorbance spectra were recorded after 100 min of photodegradation, as depicted in Fig. 5. The reduction in absorbance of each dye served as an indicator of dye degradation efficiency. Notably, 1 mg of photocatalyst loading in AC shows the highest dye degradation with the lowest absorbance intensity, as shown in Fig. 5a. Furthermore, increasing the photocatalyst loading beyond 1 mg does not significantly enhance degradation efficiency. It is expected that increasing the catalyst dose will provide more active sites for photocatalytic reactions, thereby enhancing the dye degradation efficiency. However, increases in catalyst concentration in our case led to a decrease in degradation efficiency. This phenomenon can be attributed to higher light scattering^44^ and the screening effect^45^, which masks part of the photosensitive nanocomposite. At higher catalyst concentrations, the solution becomes more turbid (see supplementary information Fig. S3), which limits light transmission and reduces the number of photons reaching the catalyst surface, thereby diminishing photocatalytic activity. Additionally, an increase in photocatalyst loading leads to particle agglomeration, reducing the effective surface area and the number of accessible active sites^44^. These effects collectively contribute to a decrease in degradation efficiency beyond the optimal loading of 1 mg, as also reported in previous photocatalytic studies^44–46^. Similarly, for CR, IC, and NR dyes, 1 mg of photocatalyst loading demonstrated optimal photodegradation efficiency, as depicted in Fig. 5b and c, and Fig. 5d, respectively. The corresponding dye degradation picture is shown in the supplementary information Fig. S3. Through a comparative analysis of degradation efficiency across various photocatalyst loadings, it is evident that a loading of 1 mg of photocatalyst is optimal for 5 mL aqueous solutions with a dye concentration of 25 ppm. Therefore, 1 mg catalyst loading for 25 ppm dye in 5 mL aqueous solution at pH 5 was considered for further studies.

#### Organic dye photodegradation performance

**Fig. 6:**
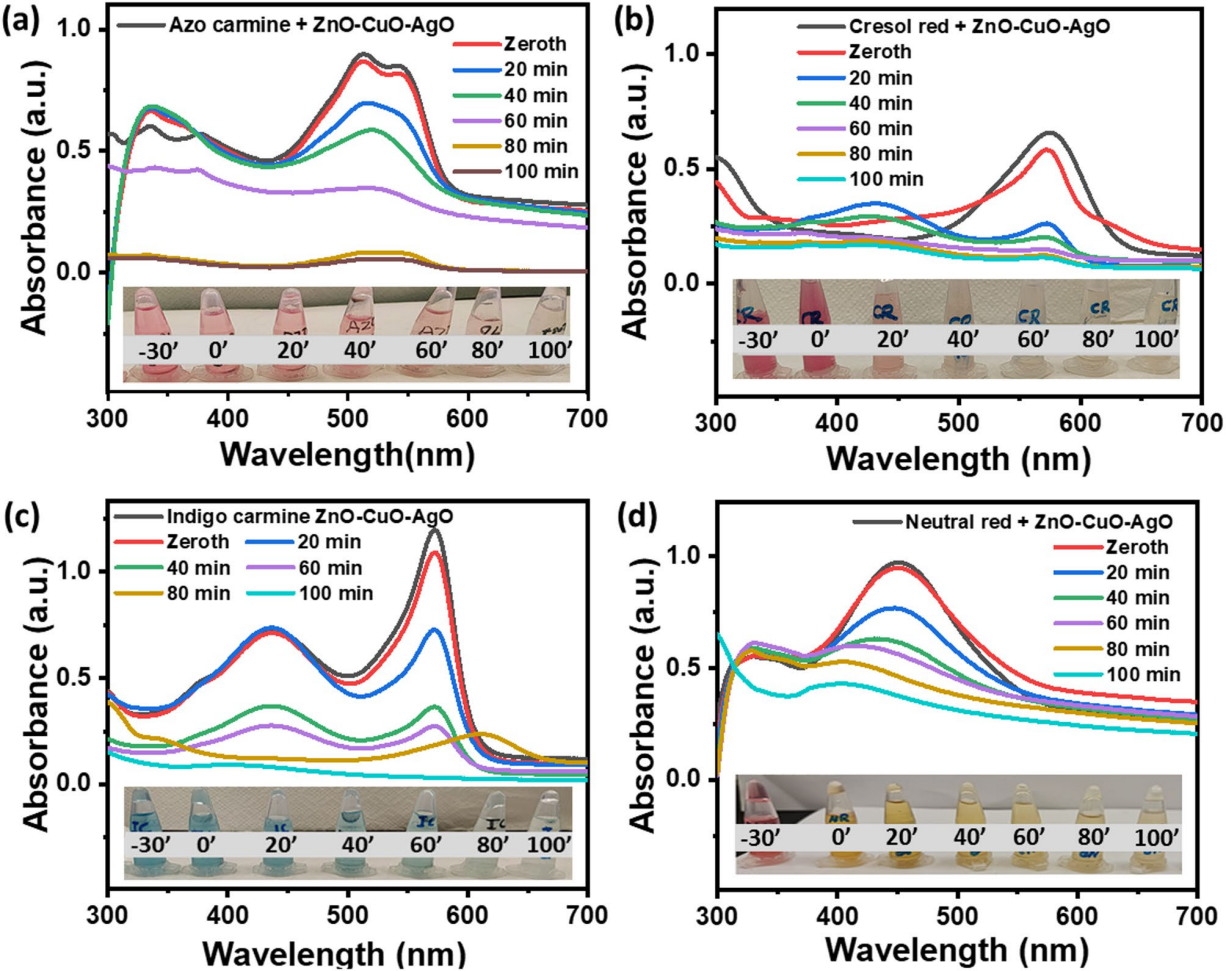
UV-visible absorbance spectra of dye concentration during photodegradation. [reaction conditions: dye concentration = 25 ppm, dye solution = 5 mL, pH = 7, photocatalyst loading = 1 mg, sunlight irradiation = 100 min]. (**a**) Azo carmine. (**b**) Cresol red. (**c**) Indigo carmine. (**d**) Neutral red.

Dye photodegradation was analyzed by monitoring the concentration of the dye solution over time using UV-visible spectroscopy. The experiment involved loading 1 mg of catalyst into 5 mL of a 25 ppm dye aqueous solution at pH 7. Absorbance measurements were taken before light exposure and at 20-minute intervals throughout the 100-minute photodegradation process. Figure 6 shows the absorbance spectra of dye concentration during photodegradation at an interval of 20 min. Figure 6a shows the absorbance spectra for the AC dye concentration during photodegradation. The absorbance spectrum of AC dye displays a characteristic peak at 514 nm. As photodegradation proceeds, the intensity of the peak at 514 nm diminishes, signifying the degradation of the AC dye molecules. The inset in Fig. 6a depicts the transformation of the AC dye solution from pink to clear during photodegradation. Similarly, Fig. 6b demonstrates the absorbance spectra during the photodegradation of CR dye, with a decrease in absorbance at a wavelength of 572 nm, indicating CR dye degradation. The inset in Fig. 6b depicts the color change of the CR dye solution from red to clear during photodegradation. Figure 6c shows the absorbance curve of IC dye during photodegradation, with a characteristic peak at 572 nm. The inset in Fig. 6c displays the color change of the IC dye solution from blue to clear during photodegradation. The photodegradation absorbance plot of NR dye is shown in Fig. 6d, exhibiting a characteristic peak at 450 nm. The picture of the NR dye solution throughout photodegradation is shown in the inset of Fig. 6d. In addition, with changes in peak intensity, shifts in the shape of the absorbance spectra occurred during dye photodegradation. This can be attributed to changes in the chemical structure of the dye molecules as they undergo chemical transformations.

**Fig. 7:**
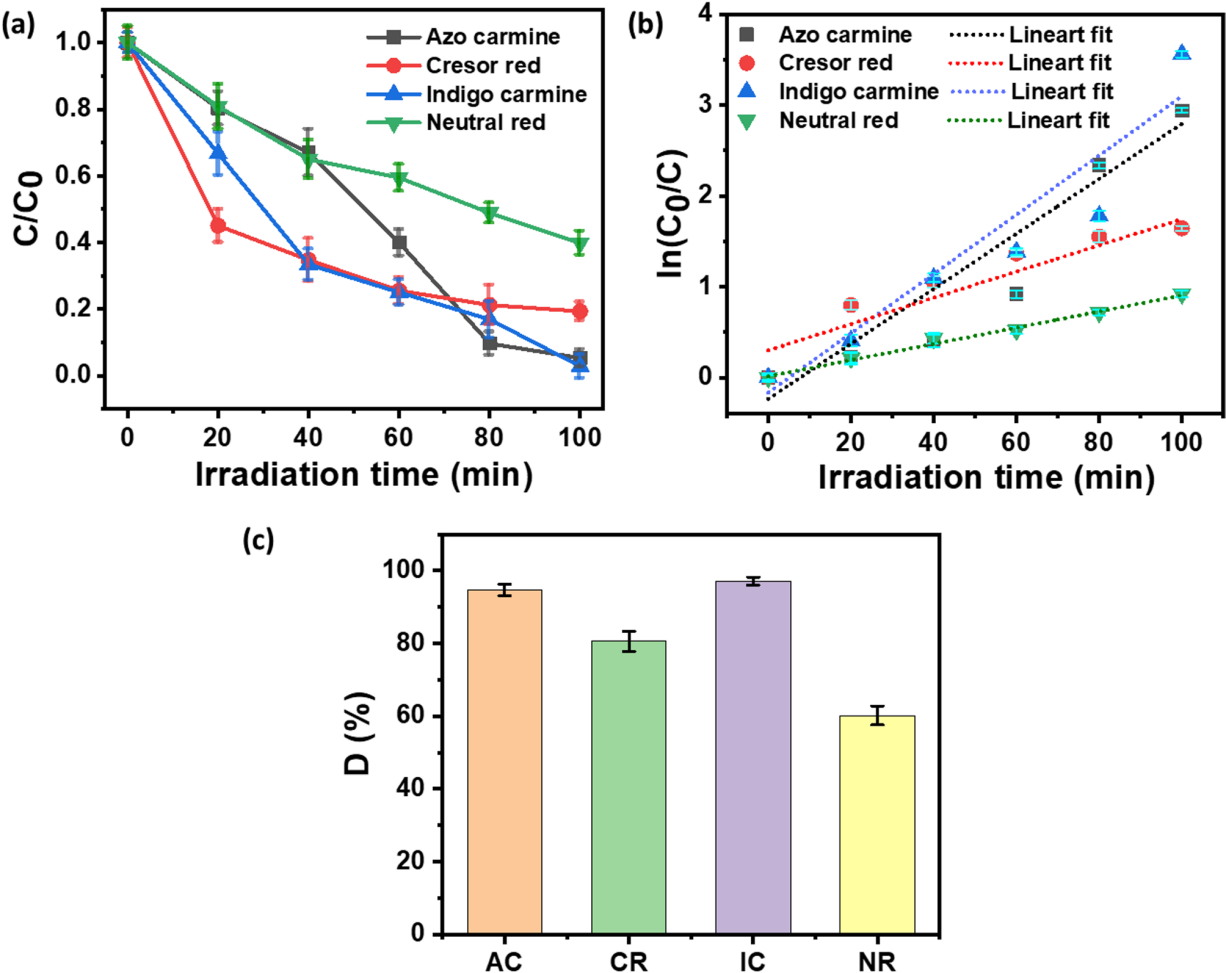
Photodegradation evaluation of AC, CR, IC and NR dye by ZnO–CuO–AgO composite [reaction conditions: dye concentration = 25 ppm, dye solution = 5 mL, pH = 7, photocatalyst loading = 1 mg, sunlight irradiation = 100 min]. (**a**) Photodegradation rates. (**b**) Corresponding reaction rate plots. (**c**) Corresponding dye degradation percentage plots.

Further, a decrease in absorbance intensity at the characteristic wavelength of the dye was used to calculate the dye degradation parameters, such as dye degradation percentage and rate kinetics. Figure 7a shows the relative photodegradation profiles of AC, CR, IC, and NR dyes with respect to time. The relative dye concentration (C/C_0_) was determined at the relative absorbance (A/A_0_) of dye characteristic peak wavelengths, where A_0_ and A and were the absorbances of dye solution at a starting time (t_0_) of photodegradation and at any time t, respectively. The characteristic peak wavelengths for AC, CR, IC, and NR were 514 nm, 572 nm, 572 nm, and 450 nm, respectively (see Fig. 6). A significant decrease in relative dye photodegradation was observed, indicating efficient dye degradation, as shown in Fig. 7a. To estimate the photodegradation rate kinetics of AC, CR, IC, and NR dyes Langmuir−Hinshelwood (L−H) model^47^ was adopted as given in Eq. 3.

2 $$\:r=-\frac{dc}{dt}=\frac{kKC}{1+KC}$$

where r is the degradation rate, k is the reaction rate constant, K is the equilibrium constant, C is the concentration of dye, and t is the sunlight irradiation time. The term kK can be termed as the apparent rate constant k_app_. The Eq. (3) was expressed as Eq. (4).

3 $$\:r=-\frac{dc}{dt}=\frac{{k}_{app}C}{1+KC}$$

The above equation could be reduced to the first-order equation for lower-concentration solutions of dye (KC ≪ 1) and presented as Eq. 5.

4 $$\:r=-\frac{dc}{dt}={k}_{app}C$$

The graph depicting ln(C_0_/C) versus t is displayed in Fig. 7b. Linear fitting was applied to the ln(C_0_/C) plot, and the resulting reaction kinetics and R^2^ values are presented in Table 1. The obtained coefficients (R^2^) for all cases exceeded 0.90, indicating a good fit of the photodegradation kinetics to the pseudo-first-order kinetic model. The calculated k_app_ values for AC, CR, IC, and NR dyes were 0.03, 0.014, 0.032, and 0.008 min^− 1^, respectively. The highest k_app_ value was observed for AC and IC dyes, suggesting the most efficient photodegradation performance for these dyes.

Further, Eq. 6 was used to compute the percentage of dye degradation.

5 $$\:D\:\left(\%\right)=\frac{\left({C}_{0}\:-\:C\right)}{{C}_{0}}\:\times\:100={k}_{app}C$$

where D is dye degradation percentage C_0_ denotes absorbance at zero-time, C denotes the absorbance at t time. Figure 7c shows the dye degradation percentage for AC, CR, IC and NR dye. The dye degradation percentage for AC, CR, IC and NR was 94.7%, 80.65%, 97.15% and 60.16%, respectively.

**Table 1:** Photodegradation parameter of the dyes.

Dye	Photodegradation rate (min^− 1^)	Photodegradation efficiency (%)	Time (min)	*R* ^2^
Azo carmine (AC)	0.03	94.7	100	0.95
Cresol red (CR)	0.014	80.65	100	0.91
Indigo carmine (IC)	0.032	97.15	100	0.92
Neutral red (NR)	0.008	60.16	100	0.99

### Quaternary dye system photodegradation performance

**Fig. 8:**
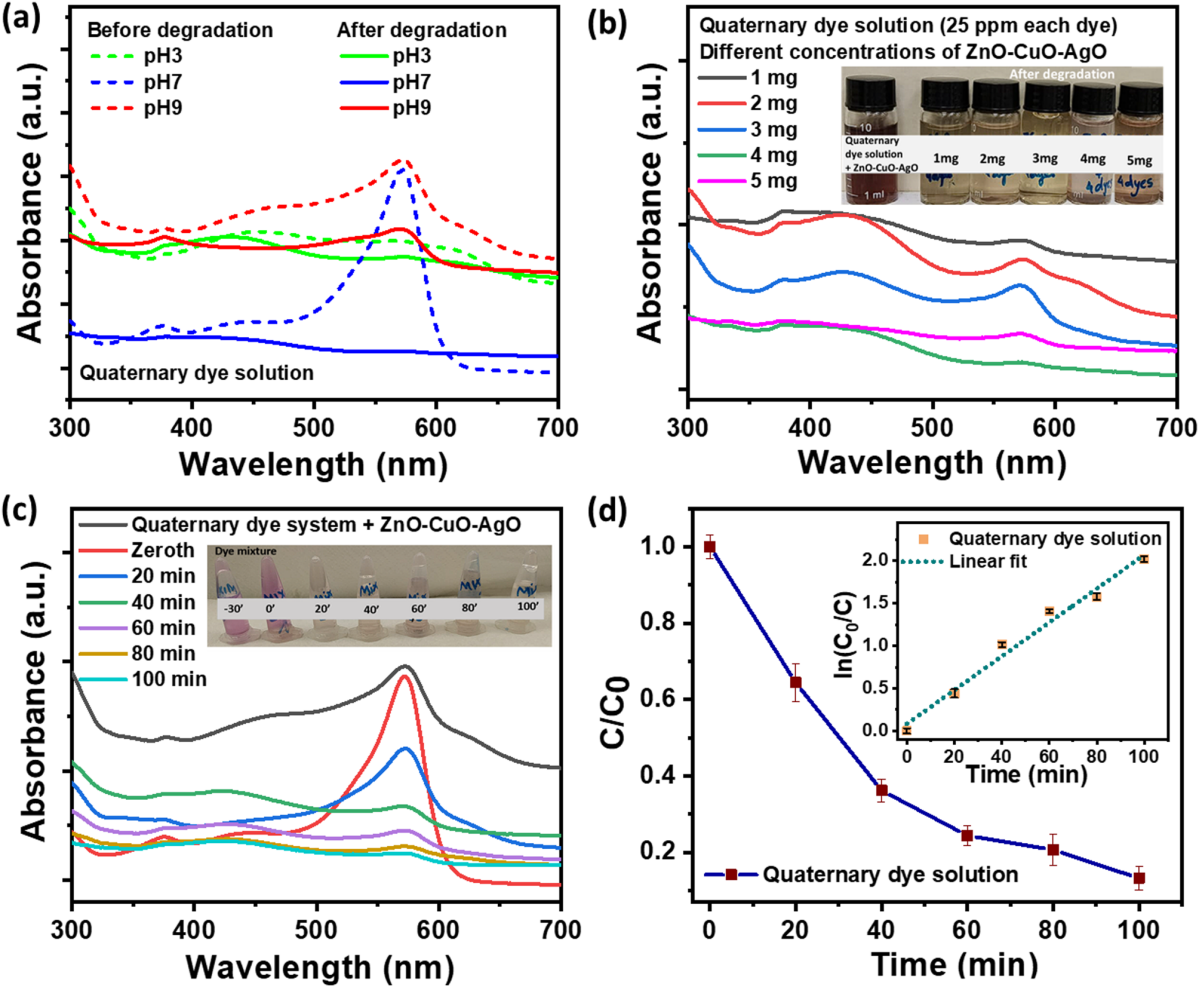
Photodegradation evaluation of quaternary dye system by ZnO–CuO–AgO composite [reaction conditions: quaternary dye system = 25 ppm AC, 25 ppm CR, 25 ppm IC, and 25 ppm NR dye, solution = 5 mL, pH = 7, photocatalyst loading = 4 mg, sunlight irradiation = 100 min]. (**a**) UV–visible absorbance spectra before and after photodegradation at different pH. (**b**) UV-visible absorbance spectra for analyzing the photocatalysis loading. (**c**) UV–visible absorbance spectra of quaternary dye system during photodegradation at an interval of 20 min (**d**) Photodegradation rates. The inset shows the corresponding reaction rate plots.

The study investigated the synergistic photodegradation efficacy of the ZnO–CuO–AgO nanocomposite in a quaternary dye solution comprising 25 ppm each of AC, CR, IC, and NR in 5 mL of aqueous solution at varying pH levels (pH 3, pH 7, and pH 9). ZnO–CuO–AgO weighing 4 mg was introduced, and the absorption spectra of the quaternary dye system were analyzed before and after 100 min of photodegradation, as depicted in Fig. 8a. Notably, the pH 7 solution demonstrated the highest degradation among the quaternary dye systems. Subsequently, to ascertain the optimal photocatalyst loading, 1–5 mg of ZnO–CuO–AgO was added to the quaternary dye system. Figure 8b illustrates the impact of photocatalyst loading on quaternary dye system photodegradation, with the 4 mg photocatalyst-loaded system exhibiting the most substantial degradation after 100 min of sunlight exposure. The results were in line with those of the previously investigated photocatalyst loading in individual dye solutions (see Fig. 5). Further analysis of the photodegradation and degradation rate of the quaternary dye system was conducted. Absorbance spectra of the quaternary dye system were recorded before exposure to sunlight and at specific time intervals during the photodegradation process using UV-visible spectroscopy. Figure 8c shows the changes in the absorbance curve of the quaternary dye system throughout the photodegradation process at an interval of 20 min. A decrease in intensity of the characteristic peak wavelength at 574 nm was observed, indicating the degradation of the quaternary dye system. The inset in Fig. 8c illustrates the color change of the quaternary dye system during photodegradation. Figure 8d depicts the relative photodegradation profile of the quaternary dye system. Subsequently, the rate of photodegradation was determined by plotting ln(C_0_/C) against time, with C_0_ representing the initial concentration of the dye mixture and C representing the concentration at each time interval (see inset of Fig. 8d). Photodegradation kinetics were assessed based on Eq. 2. The slope of the linear fit to the plot yielded the photodegradation rate, with the degradation rate constant (k_app_) calculated to be 0.03 min^− 1^. The linear fit R^2^ value was 0.97, confirming a good fit of the photodegradation kinetic model. The photodegradation percentage was calculated using Eq. 6, and a degradation efficiency of 86.72% was achieved for the quaternary dye system. Further, for the practical application of dye mixture degradation, turbine-based and carbon cloth-based prototypes powered by solar panels were developed, and quaternary dye degradation performance was examined and discussed in the subsequent section.

### Quaternary dye system photodegradation performance by turbine-based prototype

**Fig. 9:**
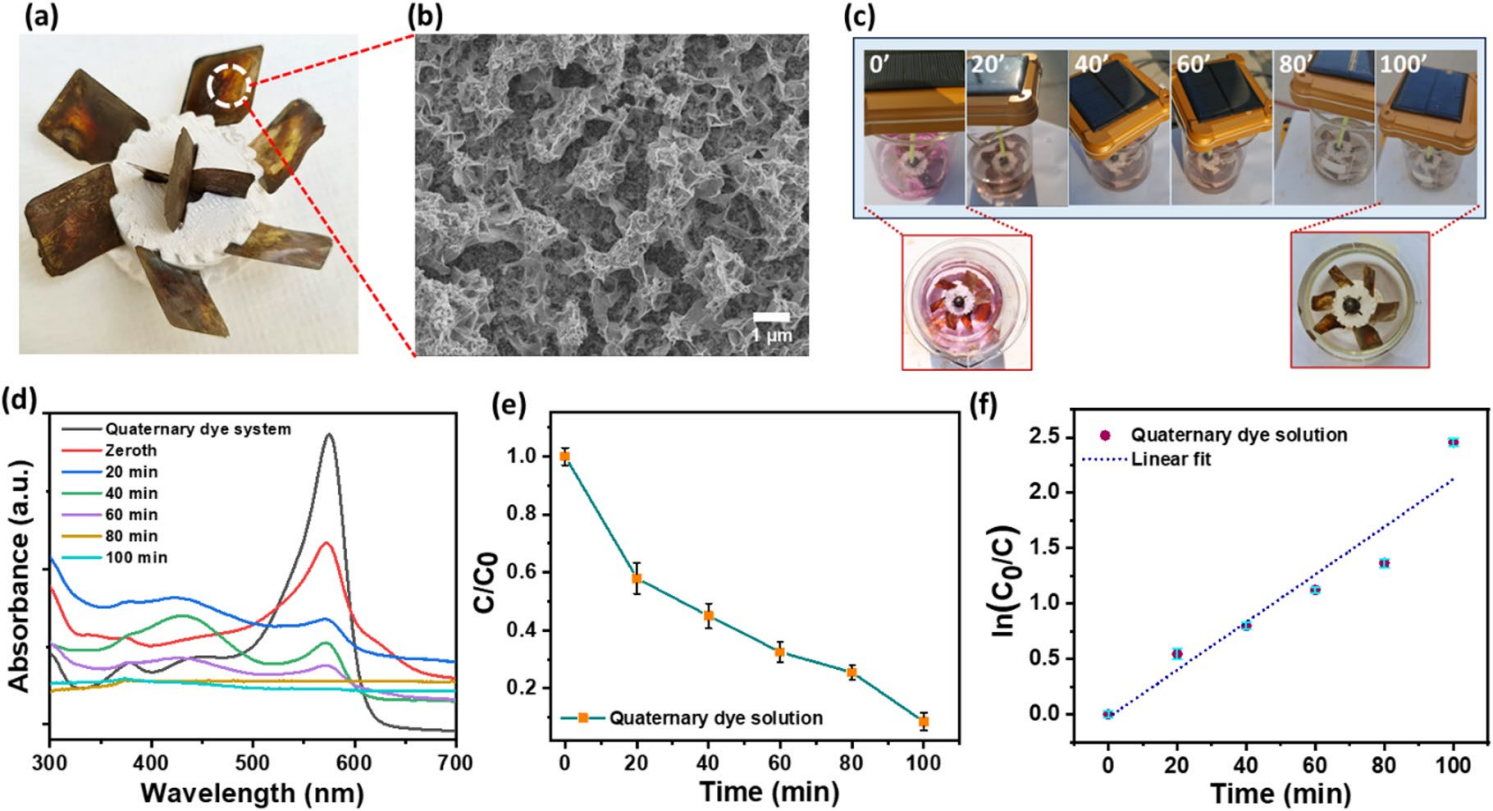
Photodegradation evaluation of quaternary dye system by ZnO–CuO–AgO decorated turbine-based prototype. [reaction conditions: quaternary dye system = 25 ppm AC, 25 ppm CR, 25 ppm IC, and 25 ppm NR dye, solution = 200 mL, pH = 7, sunlight irradiation = 100 min]. (**a**) Photograph of the 3D-printed turbine with blades decorated with ZnO–CuO–AgO nanocomposite. (**b**) SEM image of the ZnO–CuO–AgO nanocomposite decorated turbine blade. (**c**) Photograph of the quaternary dye system at various time intervals throughout the photodegradation process. (**d**) UV-visible absorbance spectra of quaternary dye system during photodegradation at an interval of 20 min. (**e**) Photodegradation rates. (**f**) Corresponding reaction rate plots.

This approach utilized a 3D-printed turbine-based prototype, integrated with solar panels to generate renewable energy for powering the photodegradation process of the quaternary dye system. Figure 9a shows the picture of the 3D-printed turbines. The turbine blades were decorated by ZnO–CuO–AgO nanocomposite and served as the photocatalyst, facilitating the degradation of the quaternary dye mixture. Figure 9b shows the SEM image of the ZnO–CuO–AgO decorated of the turbine blades. Uniform deposition of the nanocomposite was observed on turbine blades. Figure 9c presents a picture of the quaternary dye solution during different time intervals of the photodegradation process. The quaternary dye solution color diminished with an increase in photodegradation time, resulting in an almost clear solution. Figure 9d illustrates the absorbance curve of the quaternary dye system throughout the photodegradation process, with readings taken at 20-minute intervals. A decrease in the intensity of the characteristic peak at 574 nm was noted, indicating degradation of the quaternary dye system. Figure 9e presents the relative photodegradation profile of the quaternary dye system, shifting towards a linear decrease in the profile. Subsequently, the rate of photodegradation was determined by plotting ln(C_0_/C) against time, as shown in Fig. 9f. The photodegradation kinetics were evaluated using Eq. 5. The slope of the linear fit to the plot yielded the photodegradation rate, resulting in a calculated degradation rate constant of 0.021 min^− 1^. The linear fit R^2^ value of 0.95 indicates a good fit of the photodegradation kinetic model. The working video of the prototype is given in the supplementary information video V1. The turbine-based prototype achieved an impressive degradation efficiency of 91.41% for the quaternary dye system.

### Quaternary dye system photodegradation performance by cloth-based prototype

**Fig. 10:**
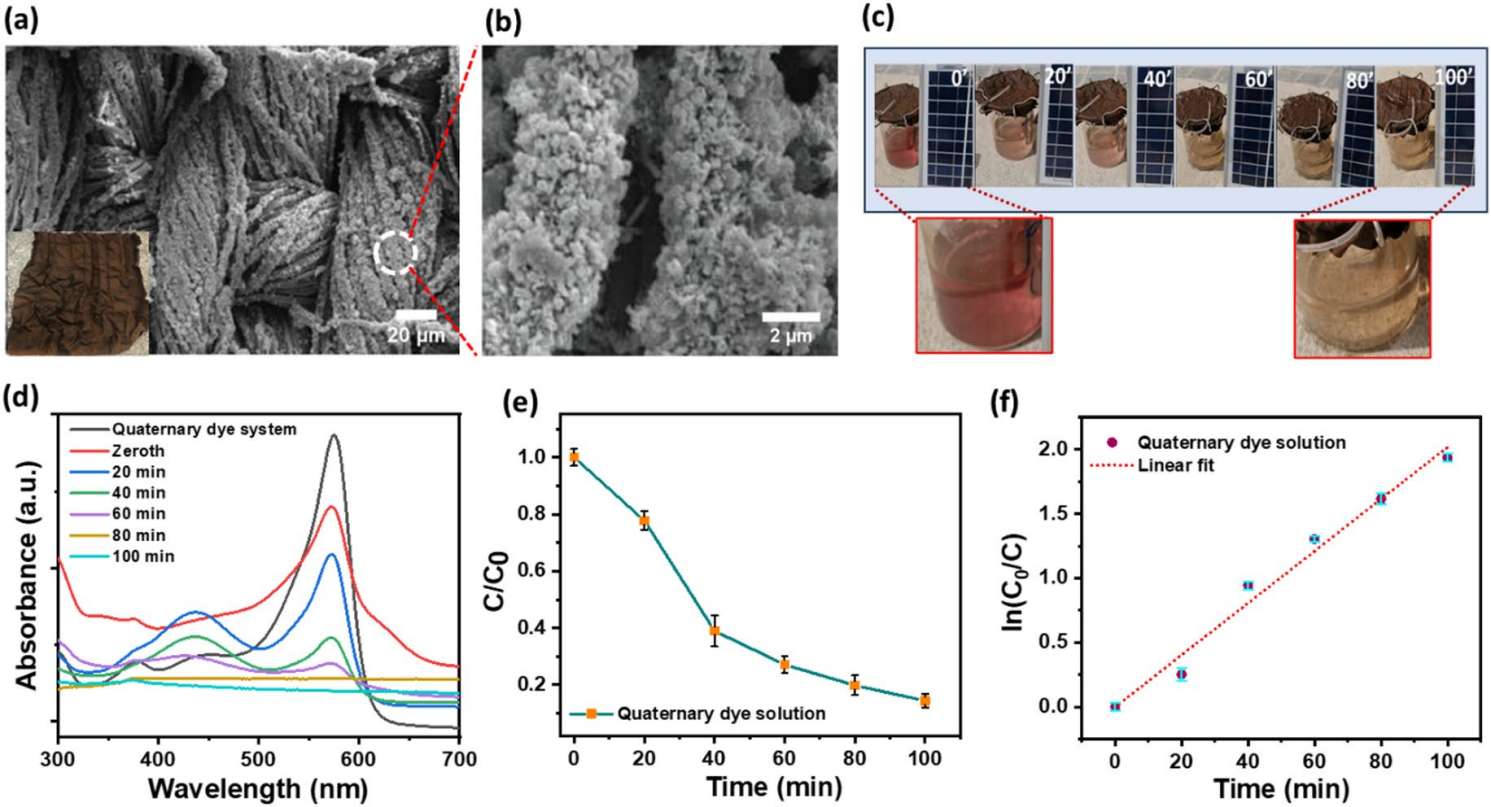
Photodegradation evaluation of quaternary dye system by ZnO–CuO–AgO decorated cloth-based prototype. [reaction conditions: quaternary dye system = 25 ppm AC, 25 ppm CR, 25 ppm IC, and 25 ppm NR dye, solution = 200 mL, pH = 7, sunlight irradiation = 100 min]. (**a**) SEM image of the ZnO–CuO–AgO nanocomposite decorated cloth. The inset shows the photograph of the ZnO–CuO–AgO cloth. (**b**) Magnified SEM image showing the dense growth of ZnO–CuO–AgO nanocomposite on the cloth fiber. (**c**) Photograph of the quaternary dye system at various time intervals throughout the photodegradation process. (**d**) UV-visible absorbance spectra of quaternary dye system during photodegradation at an interval of 20 min. (**e**) Photodegradation rates. (**f**) Corresponding reaction rate plots.

The study involved integrating a nanocomposite-decorated cloth filtration system with photodegradation capabilities to treat a quaternary dye mixture. The photocatalyst-decorated cloth served as a substrate for the photodegradation of the quaternary dye system. Figure 10a shows the surface SEM of the nanocomposite decorated on the cloth fibres. Significant coverage of the ZnO–CuO–AgO was observed on the cloth fibers. The inset of Fig. 9a shows the photograph of the nanocomposite-decorated cotton cloth. The magnified SEM cloth fiber shows the dense growth of nanocomposite, and good surface coverage is shown in Fig. 10b. Figure 10c shows a picture of the quaternary dye solution captured at various intervals during the photodegradation process. The color of the quaternary dye solution gradually faded with increasing photodegradation time, eventually resulting in a nearly clear solution. The quaternary dye solution was continuously passed through the cloth using the mini pump, powered by a solar panel. The working of the prototype is shown in the supplementary information video V2. The absorbance curve of the quaternary dye system, obtained at 20-minute intervals throughout the photodegradation process, is presented in Fig. 10d. A reduction in the intensity of the characteristic peak at 574 nm is observed, indicating the degradation of the quaternary dye system. Figure 10e illustrates the relative photodegradation profile of the quaternary dye system, showing a progressive linear decrease in the profile. Subsequently, the photodegradation rate was determined by plotting ln(C_0_/C) against time, as depicted in Fig. 10f. Photodegradation kinetics were assessed using Eq. 5. The slope of the linear fit to the plot provided the photodegradation rate, resulting in a calculated degradation rate constant of 0.02 min^− 1^. The linear fit R^2^ value of 0.98 indicated a good fit for the photodegradation kinetic model. The cloth-based prototype achieved a degradation efficiency of 88.58% for the quaternary dye system.

Table 2 compares the photodegradation parameters of ZnO–CuO–AgO composite with those reported in the literature.

**Table 2:** Comparison of photodegradation parameters ZnO–CuO–AgO with reported studies.

Catalyst	Dye	Catalyst dosage (g)	Dye concentration (ppm)	Degradation time (min)	Degradation efficiency (%)	Ref.
Co-mesoporous silica	NR	0.075	23	220	81	^48^
[Ni(2-picolinate)·H_2_O]·H_2_O	NR	0.03	10	150	82	^49^
CaO	IC	0.12	500	250	90	^50^
ZnO	IC	0.1	90	90	55	^51^
Organic acid (Oxalic acid)	CR	–	–	300	89	^52^
ZnO–CuO–AgO composite	AC, CR, IC, NR (quaternary dye system)	4 mg	25 ppm of each dye	100	86.72	Present work
3D-printed turbine decorated with ZnO–CuO–AgO	AC, CR, IC, NR (quaternary dye system)	–	25 ppm of each dye	100	91.41	Present work
Cloth decorated with ZnO–CuO–AgO	AC, CR, IC, NR (quaternary dye system)	–	25 ppm of each dye	100	88.58	Present work

The prototypes approach aimed to achieve high efficiency in dye removal while minimizing environmental impact and energy consumption. Overall, the photodegradation capability in a quaternary organic dye system was in the following order: turbine-based (91.41%) > cloth-based (88.58%) > dispersed nanocomposite (86.72%). The turbine mechanism facilitated the efficient interaction of the dye molecules with the photocatalyst and enhanced the photodegradation efficiency. As the turbine rotated, it promoted the dispersion of the photocatalyst throughout the dye solution, maximizing the contact between the photocatalyst and dye molecules. The turbine-based prototype can be applied to achieve a high reaction rate and photodegradation efficiency. The cloth-based prototype provided a sustainable and effective method for treating large volumes of dye contaminants from water sources through continuous cyclic flow. The integration of solar panels provided a sustainable approach to drive the photodegradation system.

### Reusability

**Fig. 11:**
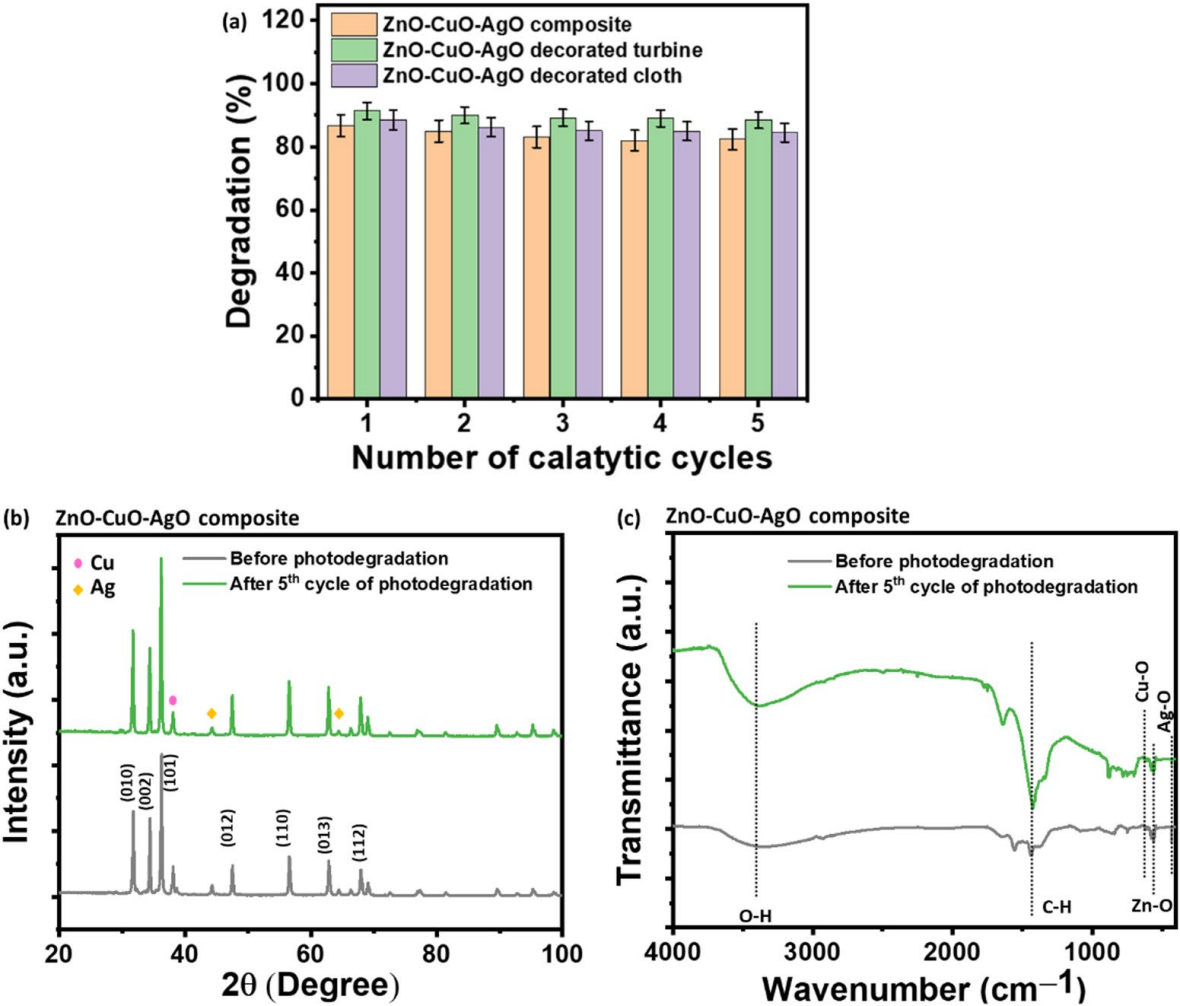
(**a**) Reusability of ZnO–CuO–AgO composite, ZnO–CuO–AgO decorated turbine and ZnO–CuO–AgO decorated cloth for degradation of quaternary dye system. (**b**) XRD plot of ZnO–CuO–AgO composite before and after five cycles of photodegradation. (**c**) FTIR curve of ZnO–CuO–AgO composite before and after five cycle of photodegradation.

Recycling experiments were conducted to evaluate the reusability and effectiveness of ZnO–CuO–AgO composite, ZnO–CuO–AgO decorated turbine, and ZnO–CuO–AgO decorated cloth after multiple uses. This process demonstrated their potential for use in continuous catalytic processes without significant loss of activity. After each cycle, the photocatalyst was separated by centrifugation at 4000 rpm for 20 min, followed by washing multiple times with DI water and finally with ethanol to remove any residual organic species or adsorbed intermediates. The ZnO–CuO–AgO decorated turbine and cloth-based prototypes were gently rinsed with DI water multiple times and finally rinsed with ethanol. The washed samples were then dried at 80 °C for 2 h before reuse. Figure 11 shows the reusability of ZnO–CuO–AgO composite, ZnO–CuO–AgO decorated turbine, and ZnO–CuO–AgO decorated cloth for the degradation of quaternary dye system. While ZnO–CuO–AgO composite and its prototype maintained their catalytic activity over multiple cycles, a slight decline in the degradation percentage of the dye system was observed after five cycles. This indicates that the reported nanocomposite and its prototype are reusable; however, some loss of efficiency may occur over time, which is a common phenomenon in catalytic materials due to surface fouling^8^. Furthermore, after five consecutive photodegradation cycles, the recovered ZnO–CuO–AgO composite was characterized using XRD and FTIR analyses. As shown in Fig. 11b, the XRD pattern confirmed that the catalyst retained its crystalline structure without the appearance of any new impurity peaks, indicating good phase stability. Similarly, the FTIR spectrum (see Fig. 11c) demonstrated that the surface functional groups of the ZnO–CuO–AgO composite remained largely unchanged after reuse. The characteristic functional group and stretching vibrations corresponding to ZnO, CuO, and AgO were evident, exhibiting only minor peak shifts attributed to repeated adsorption-desorption interactions during the photocatalytic cycles.

### Photodegradation mechanism

In order to elucidate the photodegradation mechanism of the ZnO–CuO–AgO heterostructure, the VB and CB edge positions were calculated using electronegativity theory to quantitatively verify the charge transfer mechanism. ZnO and CuO are n-type and p-type semiconductors, respectively, whereas AgO behaves as a p-type oxide with strong electron-trapping capability. Their VB and CB potential were calculated using Eqs. (7) and (8) .

6 $$\:{E}_{VB}=\chi\:-{E}_{e}+0.5{E}_{g}$$

7 $$\:{E}_{CB}={E}_{VB}-{E}_{g}$$

where $\:\chi\:$ is the absolute electronegativity, $\:{E}_{e}$ is the energy of free electrons (4.5 eV), and $\:{E}_{g}$ is the band gap. The average of the electron affinity and first ionizing energy was used to calculate the electronegativity^53^.

**Fig. 12:**
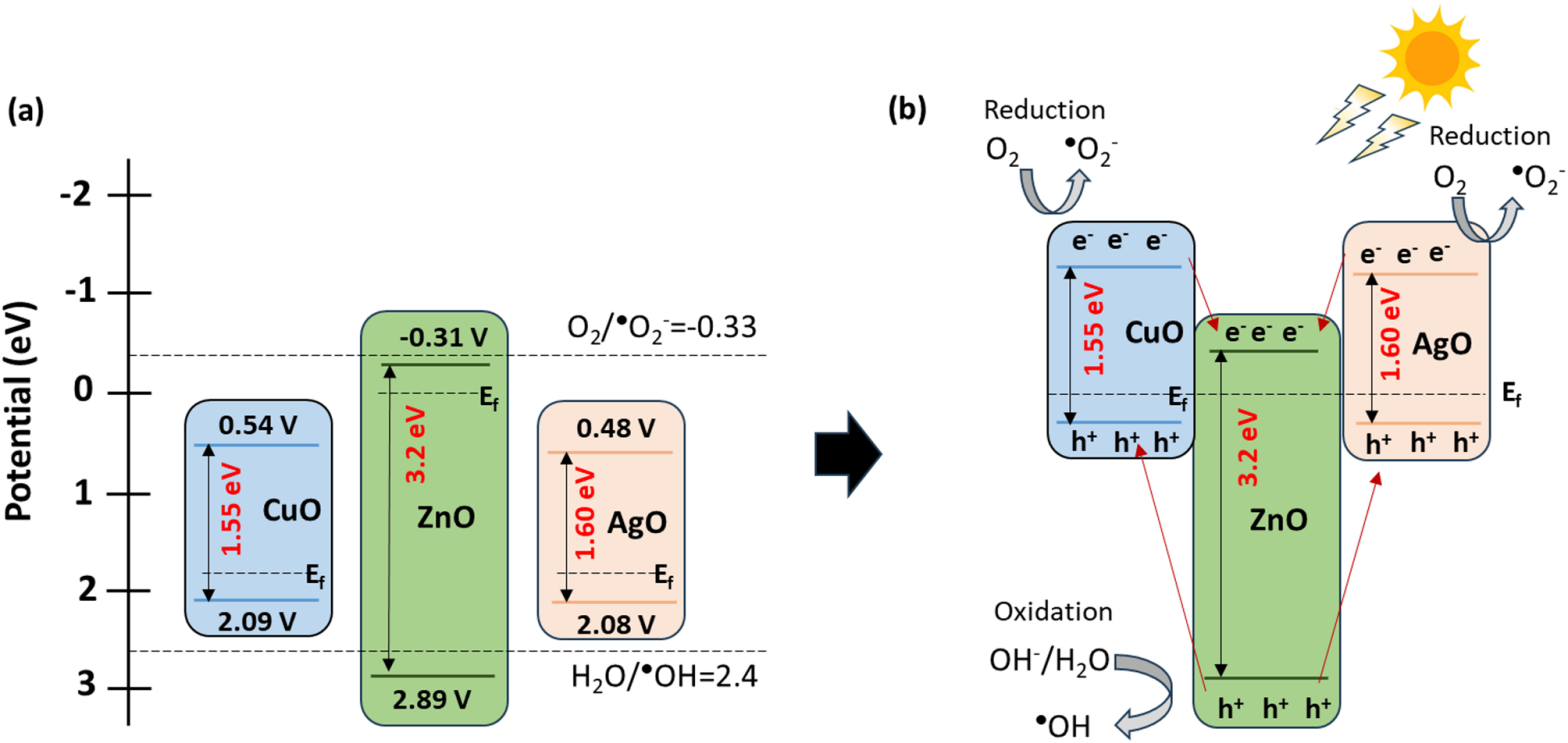
(**a**) Schematic energy band diagrams of the catalysts. (**b**) Schematic representing tuning of band structure and a photocatalytic mechanism of ZnO–CuO–AgO heterostructure for the photodegradation of AC, CR, IC, and NR dye.

Figure 12a shows the determined VB/CB edge position for CuO (+ 2.09/+0.54 eV), ZnO (+ 2.89/–0.31 eV), and AgO (+ 2.08/+0.48 eV)^53^. Figure 12b shows the band tunning and proposed photodegradation mechanism and proposed photodegradation mechanism. The photodegradation of dye by ZnO–CuO–AgO composite is explained by Eqs. 9–14^22^. When exposed to visible light, ZnO, CuO and AgO generate electron-hole (e⁻/h⁺) pairs^54^. Due to the band alignment, electrons in the CB of CuO and AgO readily transfer to the CB of ZnO, while holes in the VB of ZnO migrate to the VB of CuO and AgO. The heterojunction formation between the n-type ZnO/p-type CuO and n-type ZnO/p-type AgO suppresses the recombination and prolongs the lifetime of photogenerated pairs. As a result, electrons on the CuO, AgO surface reduce dissolved O₂ to superoxide radicals (˙O₂⁻). Part of ˙O₂⁻combines with H^+^ to form H_2_O_2,_ which could be decomposed into **˙**OH. In addition, the holes in the VB of ZnO transform H_2_O molecules or surface hydroxyl groups to ˙OH radicals. The ˙O₂⁻, ˙OH radicals, and holes will be responsible for decomposing the dye molecules effectively.

8 $${\text{ZnO}}\:\left({e}^{-}+\:{h}^{+}\right)+\text{CuO}\:\left({e}^{-}+\:{h}^{+}\right)+\:\text{AgO}\:\left({e}^{-}+\:{h}^{+}\right)\underrightarrow{h\upsilon\:}\:\text{ZnO}\:\left({e}^{-}+\:{e}^{-}\right)+\:\text{CuO}\:\left({h}^{+}+\:{h}^{+}\right)+\:+\:\text{AgO}\:({h}^{+}+\:{h}^{+})$$

Oxidation

9 $$\:{h}^{+}+\:\text{H}_{2}\text{O}\:\to\:\cdot{}\text{OH}+\text{{H}}^{+}/{h}^{+}+\:+\: \text{{OH}}_{ads}^{-}\:\to\:\:\cdot{}\text{OH}\:$$

10 $$\:2\cdot{}\text{OH}\:\to\:\:\text{H}_{2}\text{O}_{2}+\text{O}_{2}\:$$

11 $$\:\text{H}_{2}\text{O}_{2}+\:{e}^{-}\to\:\cdot{} \text{OH}+\text{OH}^{-}$$

Reduction

12 $$\:{e}^{-}\:+\:\text{O}_{2\:ads}\to\:\:\cdot \text{O}_{2}^{-}$$

13 $$\:{\cdot{} \text{O}}_{2}^{-}+\:{H}^{+}\to\:\text{HO}_{2}^{\cdot{}}$$

Degradation products

14 $$\:{h}^{+}/{\cdot{}\text{O}}_{2}^{-}/\cdot{}\text{OH}+dye\:\to\:\:\text{H}_{2}\text{O}+\text{CO}_{2}\:$$

**Fig. 13:**
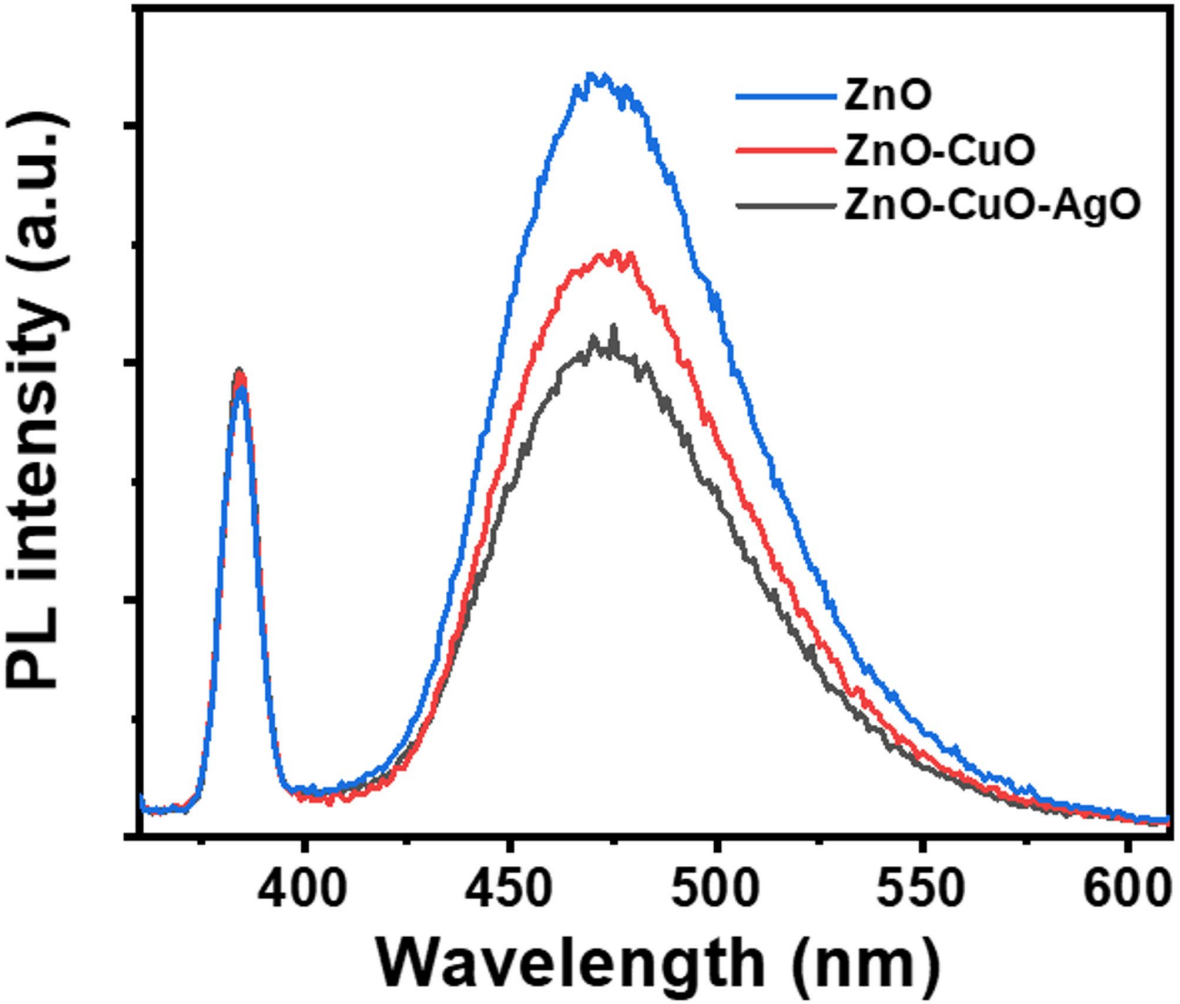
Photoluminescence (PL) spectra of ZnO–CuO–AgO composite.

Figure 13 shows the PL spectra of ZnO, ZnO-CuO, and ZnO–CuO–AgO obtained at an excitation wavelength of 325 nm. The PL analysis provides clear evidence of the role of heterojunctions in suppressing charge recombination and enhancing photocatalytic activity. Pure ZnO exhibits a strong near-band-edge UV emission at ~ 380 nm along with a broad visible emission band (450–600 nm), both of which indicate a high probability of electron-hole recombination. These emissions typically arise from oxygen vacancies, zinc interstitials, and defect levels within the ZnO lattice^55^. Upon coupling with CuO, the PL intensity decreases noticeably due to the formation of a p-n heterojunction that promotes directional charge transfer, with electrons migrating into ZnO and holes into CuO, thereby reducing radiative recombination. This quenching effect is even more pronounced in the ZnO–CuO–AgO ternary composite, where AgO nanoparticles act as electron reservoirs and interfacial mediators, further extending carrier lifetimes and suppressing recombination across both UV and visible regions. The progressive quenching from ZnO to ZnO-CuO to ZnO–CuO–AgO demonstrates the effectiveness of heterojunction engineering in improving charge separation, which directly correlates with enhanced photocatalytic dye degradation efficiency.

## Conclusion

In summary, ZnO–CuO–AgO nanocomposite was successfully synthesized using the hydrothermal method. The formation of the heterostructure was confirmed by XRD and FTIR spectroscopy. The morphology of the nanocomposite was analyzed by TEM analysis. The photodegradation of azo carmine (AC), cresol red (CR), indigo carmine (IC), and neutral red (NR) dye was evaluated. The nanocomposite exhibited the best dye degradation in pH 7 aqueous solution with optimal photocatalyst loading of 0.2 gL^− 1^ for 25 ppm dye solution. The reaction rate values for AC, CR, IC, and NR dyes were 0.03, 0.014, 0.032, and 0.008 min^− 1^, respectively. The dye degradation percentage for AC, CR, IC and NR was 94.7%, 80.65%, 97.15% and 60.16%, respectively. The study further investigated the synergistic photodegradation efficacy of the ZnO–CuO–AgO nanocomposite in a quaternary dye solution. The photodegradation rate was estimated to be 0.019 min^− 1^, and a degradation efficiency of 86.72% was achieved for the quaternary dye system. Additionally, the development of ZnO–CuO–AgO decorated turbine-based and cloth-based prototypes for practical application in quaternary dye mixture degradation demonstrated promising results, achieving high degradation efficiencies of 91.41% and 88.58%, respectively. These findings underscore the potential of the developed prototypes for sustainable and efficient dye wastewater treatment.

## Supplementary Information

Below is the link to the electronic supplementary material.

Supplementary Material 1

Supplementary Material 2

Supplementary Material 3

Supplementary Material 4

## Data Availability

The data supporting the findings of this study will be made available on request from the corresponding author.
