# Supplementary material for: A prototype integrated approach for sustainable treatment of organic dyes system using ZnO–CuO–AgO heterostructure as photocatalyst
Source: Sci Rep. 2025 Dec 11;16:380. doi: 10.1038/s41598-025-29850-1 (PMC12770352; doi:10.1038/s41598-025-29850-1)
Supplement: Supplementary file 1 — Supplementary Material 1 [file 41598_2025_29850_MOESM1_ESM.docx]

**A Prototype Integrated Approach for Sustainable Treatment of Organic Dyes System Using ZnO-CuO-AgO Heterostructure as Photocatalyst**

Muni Raj Maurya^1^, Sumalatha Bonthula^1^, John-John Cabibihan^2,^ *, Alanood Alsafri^3^, Omar Al Sakka Amini^3^, Noora Noora^3^, Kishor Kumar Sadasivuni^1, 2,^ *

*^1^Center for Advanced Materials, Qatar University, PO Box 2713 Doha, Qatar.*

*^2^Department of Mechanical and Industrial Engineering, Qatar University, PO Box 2713, Doha, Qatar*

*^3^College of Engineering,* *Qatar University, PO Box 2713, Doha, Qatar*

*^*^Corresponding author:* [kishorkumars@qu.edu.qa](mailto:kishorkumars@qu.edu.qa) (K.K.S), [john.cabibihan@qu.edu.qa](mailto:john.cabibihan@qu.edu.qa) (J.J.C.)


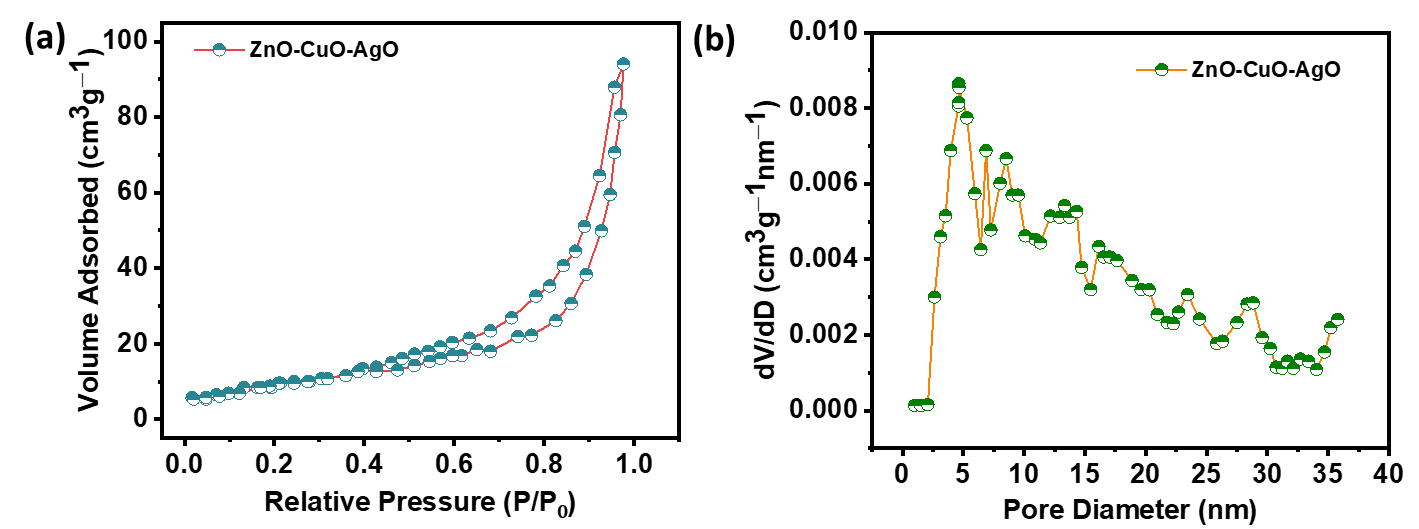


**Fig. S1.** (a) BET curve of ZnO-CuO-AgO. (b) Pore size distribution of ZnO-CuO-AgO.


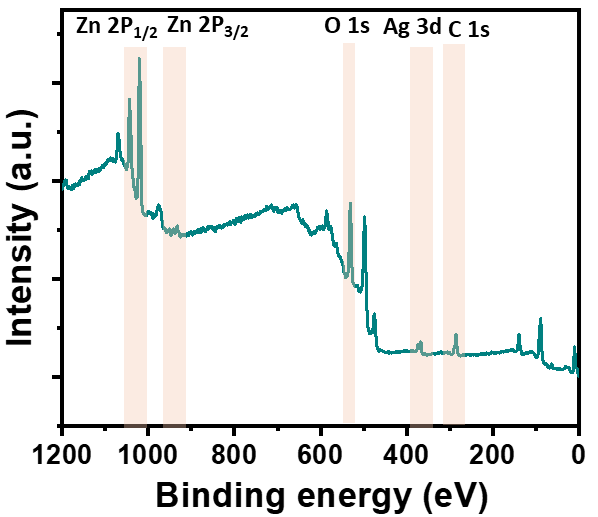


**Fig. S2.** XPS spectrum of ZnO-CuO-AgO


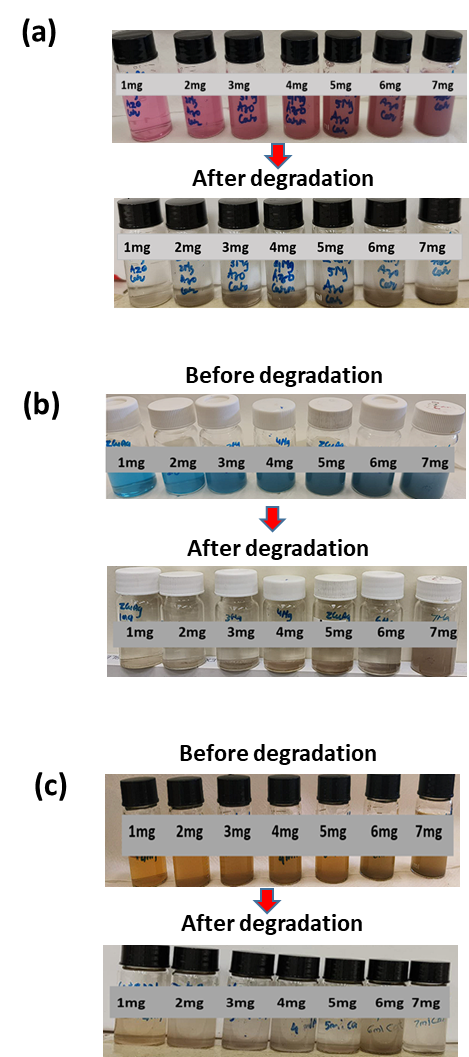


**Fig. S3.** Photograph dye before and after 100 min of photodegradation with different photocatalyst loading. (a) Azo carmine (b) Indigo carmine. (c) Neutral red.
