# Supplementary material for: A prototype integrated approach for sustainable treatment of organic dyes system using ZnO–CuO–AgO heterostructure as photocatalyst
Source: Sci Rep. 2025 Dec 11;16:380. doi: 10.1038/s41598-025-29850-1 (PMC12770352; doi:10.1038/s41598-025-29850-1)
Supplement: Supplementary file 4 — Supplementary Material 4 [file 41598_2025_29850_MOESM4_ESM.docx]

**Supplementary information Video 1**

***Figure title:*** Quaternary dye system photodegradation using 3D printed prototype loaded with ZnO-CuO-AgO nanocomposite.

***Legend:*** Turbine prototype

**Supplementary information Video 2**

***Figure title:*** Quaternary dye system photodegradation using cloth loaded with ZnO-CuO-AgO nanocomposite under cyclic flow of the dye-contaminated water.

***Legend:*** Cloth prototype
